# Friction and Cartilage Wear in Hemiarthroplasty: A Systematic Review of Key Influencing Factors

**DOI:** 10.3390/lubricants14010018

**Published:** 2025-12-31

**Authors:** Victoria P. Marino, Francesca De Vecchi, Dominik J. Federl, Landon M. Begin, Afton K. Limberg, Douglas C. Moore, Joseph J. Crisco, Douglas W. Van Citters, Markus A. Wimmer

**Affiliations:** 1 Department of Orthopedic Surgery, Rush University Medical Center, Chicago, IL 60612, USA; 2 Department of Biomedical Engineering, University of Illinois at Chicago, Chicago, IL 60607, USA; 3 Institute for Biology, Engineering, and Medicine, Brown University, Providence, RI 02912, USA; 4 Thayer School of Engineering, Dartmouth College, Hanover, NH 03755, USA; 5 Department of Orthopaedics, The Warren Alpert Medical School, Brown University, Providence, RI 02903, USA

**Keywords:** hemiarthroplasty, coefficient of friction, cartilage wear, materials, in vitro, contact stress, velocity

## Abstract

Hemiarthroplasty addresses joint damage confined to one side, preserving native cartilage and bone, but accelerated degeneration of the opposing cartilage can compromise outcomes. This systematic review should clarify whether coefficient of friction (COF) reliably predicts cartilage wear when evaluating hemiarthroplasty bearing materials (HBMs). Thirty in vitro studies reporting both outcomes were identified. Data were extracted on COF, wear, and testing parameters, and wear was standardized using a 0–4 rubric to enable cross-study comparison. Three analytical approaches were applied: linear model fits, Pearson’s correlations, and predictive modeling. Reported COFs increased significantly with testing time, while contact stress and sliding velocity showed variable associations with COF. Predictive models for cobalt–chromium (CoCr), the most studied HBM, showed moderate fit, suggesting that mechanical parameters explain only part of COF variability. For wear, linear models showed poor fit with COF, but correlations indicated positive associations with contact stress. Inconsistent effects of velocity and distance were found. Predictive models explained little variability. Together, these findings suggest that outcomes are strongly influenced by testing conditions, lubricants, and HBM selection, and COF alone is an unreliable predictor of cartilage wear in an experimental setting.

## 1. Introduction

Hemiarthroplasty is a surgical procedure in which only one side of a joint is replaced with a prosthetic implant, preserving the opposing native cartilage and bone. This approach is most commonly employed in the hip and shoulder joints for conditions such as displaced femoral neck fractures, osteonecrosis, and localized joint degeneration [1–6]. By preserving natural cartilage, hemiarthroplasty offers the potential advantages of reduced operative time, preservation of bone stock, and minimized disruption to surrounding soft tissues. The long-term success of hemiarthroplasty depends to a large degree on the ability of the hemiarthroplasty bearing material (HBM) to interact favorably with native cartilage [7–11]. A key concern is accelerated degeneration of the opposing cartilage that compromise clinical outcomes [12–15]. Currently, there are relatively few commonly used HBMs and none are optimal.

**Figure 1.**
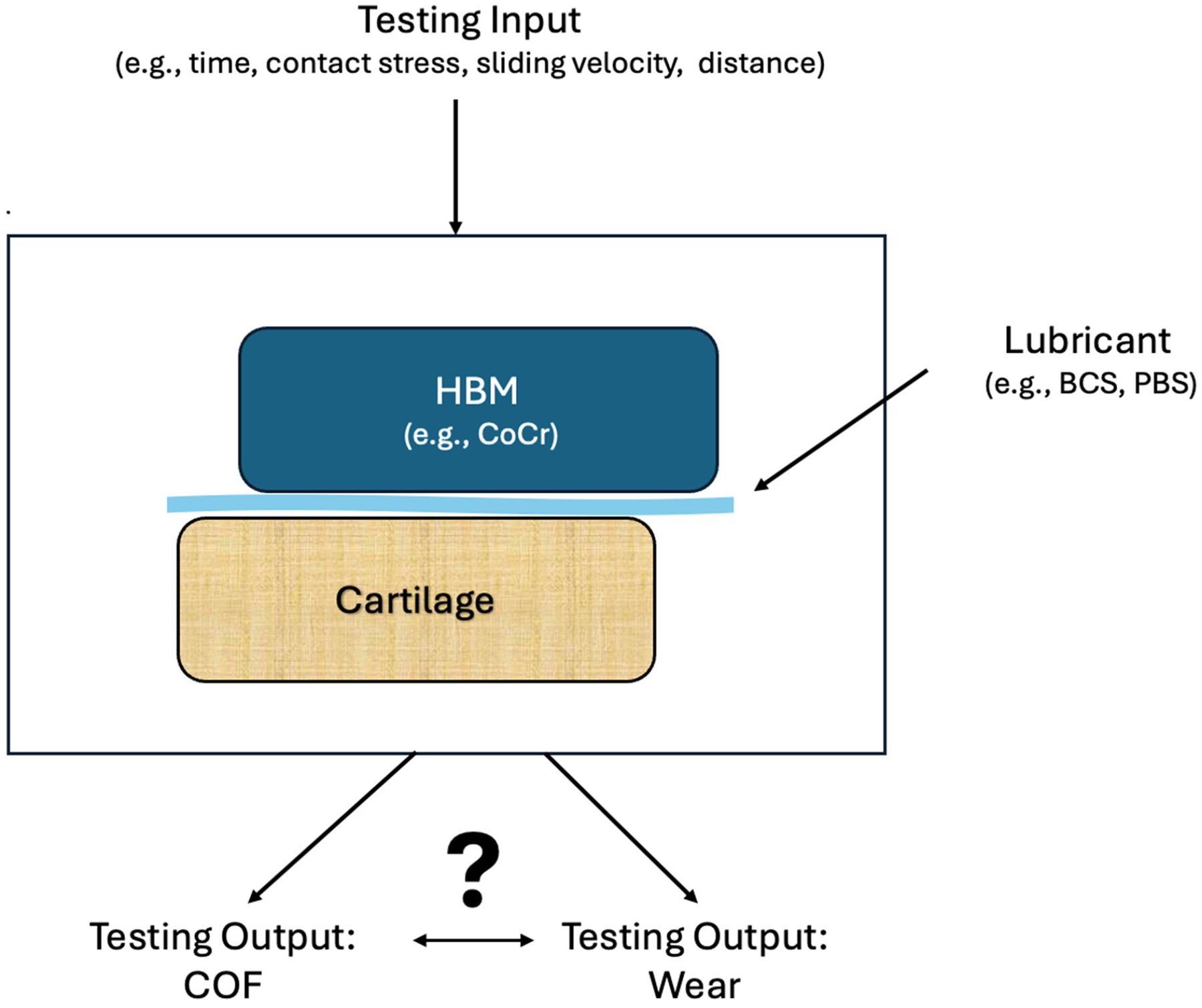
Conceptual schematic illustrating the general experimental configuration used to study cartilage–hemiarthroplasty bearing material (HBM) interactions across the 30 studies included in this systematic review. Specimen geometries and sizes varied substantially between studies; therefore, the schematic is not drawn to scale and is intended to illustrate general testing concepts rather than quantitative dimensions. Study-specific geometries and dimensions are reported in the Supplementary Data. The schematic depicts a cartilage specimen (tan) articulating against an HBM (blue), such as cobalt–chromium (CoCr), under lubrication with bovine calf serum (BCS) or phosphate-buffered saline (PBS) (light blue). Test parameters—including contact stress, sliding velocity, sliding distance, and testing duration—are precisely controlled, with outcome measures comprising the coefficient of friction (COF) and cartilage wear. The question mark indicates uncertainty, highlighting the central focus of this review: the relationship between COF and cartilage wear.

Reliable preclinical testing protocols are needed to identify implant materials that articulate favorably with cartilage and better predict implant longevity. In vitro testing typically involves controlling key parameters such as HBM and lubricant selection, applied load (contact stress), sliding velocity, sliding distance, and test duration. Outputs generally include measurements of coefficient of friction (COF) and cartilage wear. The typical experimental setup used for these measurements is illustrated in Figure 1. This setup provides the framework for investigating whether COF measurements are related to subsequent cartilage degradation, addressing the central question of this review.

In September 2024, the ISO Standards Committee for Bone and Joint Replacement convened a workshop to examine current approaches in hemiarthroplasty and to initiate discussions on standardizing methodologies for evaluating the mechanical and tribological behavior of soft tissues. A key question that emerged in that workshop, which remained unresolved due to insufficient evidence, was whether friction between HBMs and cartilage could serve as reliable predictors of cartilage wear in vivo. In contrast to wear testing, friction analysis would allow much shorter testing times, thus simplifying cartilage handling procedures and avoiding the risk of tissue infection and decomposition. While high friction can contribute to wear, it is not the sole determining factor. Other factors like surface load, sliding distance, material properties (hardness, surface finish), and lubrication also play significant roles in wear. Although increased friction often accompanies surface degradation, it has not been shown to directly correlate with the severity or progression of wear, including cartilage damage [16]. Different HBMs can exhibit similar friction against cartilage but have vastly different wear behaviors. Thus, the question remains, do COF measurements accurately reflect the risk or extent of cartilage wear?

A recent scoping review of in vitro studies identified substantial variability in reported COF for the same HBM across different studies [17]. For example, COF values for cobalt-chromium alloy against cartilage have been reported to range from 0.02 to 0.57—a nearly 30-fold difference. This level of variability raises inevitable questions regarding test conditions, measurement protocols, and the interpretation of tribological findings, particularly in terms of their ability to predict cartilage damage in vivo, such as tissue wear. Similarly in animal models, the diversity of testing methodologies, inconsistent outcome metrics, and the lack of comparative evaluations across HBMs limits the ability to identify the best practice for evaluating HBM performance [18].

A key challenge in designing meaningful in vitro studies is the need to carefully define and control a range of test parameters. These include the selection of HBM and lubricant, the applied load (or contact stress), the motion path of the testing apparatus, the sliding velocity, the sliding distance, and the duration of testing. Each of these parameters can substantially influence frictional behavior and, ultimately, the cartilage wear. Therefore, standardization—or at least detailed reporting—of these parameters is essential for interpreting results and comparing findings across studies.

Hemiarthroplasty bearing materials (HBMs) exhibit wide variability in tribological performance, yet it remains unclear how frictional behavior relates to cartilage damage under simulated joint conditions. To address this gap, this systematic review evaluated 30 in vitro studies that modeled hemiarthroplasty contact, with a focus on three core questions: (1) which factors influence the coefficient of friction (COF) in HBMs, (2) which factors determine cartilage wear, and (3) how COF and wear are related. These analyses included consideration of key experimental factors such as contact stress, sliding velocity, sliding distance, test duration, and lubricant. COF, wear outcomes, and testing conditions were extracted across studies. Because cartilage wear outcomes were reported inconsistently, a standardized semi-quantitative scoring system was developed to enable cross-study comparison. Relationships between COF, test parameters, wear, and HBMs were then evaluated using model fitting, correlation analyses, and predictive modeling, providing a framework to quantify how frictional behavior and experimental conditions may predict cartilage degradation. The goal of this review was to enhance the interpretation of tribological data in hemiarthroplasty models and to inform the development of more predictive and standardized preclinical evaluation methods for HBMs. Ultimately, such improvements facilitate better translation of in vitro findings to in vivo outcomes relevant for predicting clinical performance. This systematic review evaluated 30 studies to investigate the relationships between coefficient of friction (COF), wear, and key testing parameters—contact stress, sliding velocity, sliding distance, and testing time—across various hemiarthroplasty bearing materials (HBM) and lubricants.

## 2. Materials and Methods

### 2.1. Systematic Literature Search and Study Selection

This systematic review was conducted following the Arksey and O’Malley framework [19], a structured methodology for scoping and systematic reviews that provides clear steps for defining research questions, identifying relevant studies, selecting studies based on predefined criteria, charting and extracting data, and synthesizing and reporting results. The review protocol was registered in PROSPERO (ID: 1235851), and the review was conducted and reported in accordance with Preferred Reporting Items for Systematic Reviews and Meta-Analyses (PRISMA) guidelines [20], ensuring methodological transparency and reproducibility. Detailed search strings and study selection flow diagrams are provided in the Supplementary Material.

Database searches were performed in December 2024 across PubMed, Scopus, Google Scholar, and the Cochrane Library. The search strategy, developed in collaboration with research librarians from Rush University and the University of Illinois at Chicago, was designed to capture studies reporting coefficient of friction (COF) and cartilage wear in hemiarthroplasty contexts (Table 1) and was refined based on prior work [17,18]. Retrieved records were imported into Covidence, an online platform that facilitates systematic review screening, data extraction, and collaboration. Duplicates were removed, and titles and abstracts were screened to identify studies that directly examined HBM–cartilage interactions. Only in vitro studies were ultimately included. Hemiarthroplasty bearing materials (HBMs) were defined as synthetic or metallic implant surfaces interacting with cartilage under experimental conditions. Exclusion criteria included non-English articles, conference abstracts, and non–peer-reviewed sources. Full texts of eligible studies were reviewed, and relevant data were extracted in a standardized manner.

### 2.2. Data Extraction

Data extracted from the included studies included COF, cartilage wear outcomes, and key experimental input parametersincluding contact stress, sliding velocity, sliding distance, test duration, and lubricantwere independently collected by authors V.P.M., D.J.F., F.D.V., L.M.B. and A.K.L. If necessary, discrepancies were resolved through discussion with M.A.W. Table 2 summarizes the types of data extracted.

Cartilage wear was broadly defined to include macroscopic surface damage (fibrillation, fissures, or erosion), microscopic and histological changes (matrix depletion, proteoglycan loss, or cell death), and structural alterations such as thickness reduction or permanent deformation. To enable cross-study comparisons, a standardized 0–4 scoring rubric was developed (Table 3), where 0 represented no wear and higher scores indicated progressively greater wear. Rubric criteria included macroscopic appearance, microscopic and histological findings, surface roughness, mechanical recovery, and deformation. Qualitative descriptions and representative outcomes from each study were mapped to this rubric, with all assessment modalities weighted equally. Descriptive outcomes were ranked relative to other groups using this rubric framework within each study, rather than being normalized across studies. All five authors independently assigned scores, and the average was retained for analysis. Although biochemical markers are used in other studies, none of the included studies reported or relied on biochemical assays to assess cartilage degradation. The extracted COF values and wear scores were subsequently used for correlation analyses, model fitting, and predictive modeling to evaluate relationships between material type, test parameters, and cartilage degradation.

### 2.3. Data Analysis

Data were aggregated in Microsoft Excel with organization, visualization, and statistical analysis performed using MATLAB R2024b. When a study included multiple experimental setups, replicates, or HBM types, each outcome was treated as a separate data point. Similarly, for studies reporting multiple experimental groups, every individual trial within each group was considered an independent data point for statistical analyses. A minimum of three data points from a given experiment was required for inclusion in correlation or regression analyses, regardless of whether all data originated from a single study. In cases where studies reported only average COF values without access to individual data points, these averages were included only in analyses that used average COF. They were not included in the linear mixed-effects model fits, which required discrete individual time-point measurements. Given these inclusion criteria, some HBM–lubricant combinations were represented by relatively few observations (n ≤ 4), and correlations derived from these subsets should therefore be interpreted with caution due to limited statistical power. Therefore, to maintain transparency, all such observations are explicitly included and tabulated in this review.

### 2.4. Risk of Bias Assessment Approach

Risk of bias for all included in vitro studies was evaluated using the QUIN Tool [21], which consists of 12 criteria assessing methodological quality. Each criterion was rated as adequately specified (2 points), inadequately specified (1 point), not specified (0 points), or not applicable (excluded from scoring). Two reviewers independently applied the QUIN Tool to each study. Discrepancies between reviewers were resolved through discussion, and when consensus could not be reached, a third reviewer adjudicated. For each study, the total score was calculated by summing the points across all applicable criteria. The final risk-of-bias percentage was computed using the formula [21]:

(1) Finalscore=(Totalscore×100)/(2×numberofapplicablecriteria)

Based on this percentage, studies were categorized as having low (>70%), medium (50–70%), or high (<50%) risk of bias.

#### 2.4.1. Coefficient of Friction (COF)

Measurements of the COF were collected at three time points: 600, 1800, and 3600 s to capture the time-dependent tribological behavior of cartilage, reflecting early, intermediate, and longer-term changes in friction during the experiments. At each time point, the coefficient of variation (CV) was calculated for each HBM to quantify variability in frictional behavior. To assess temporal trends in COF while accounting for inter-HBM variability, a linear mixed-effects model was fitted, with HBM included as a random effect and time as a fixed effect. The model provided estimates of the overall slope, intercept, 95% confidence intervals (CI), and R^2^, and reported *p*-values for fixed effects. For more detailed analyses, linear regression models were fitted for each HBM-lubricant combination with at least three measurements to evaluate HBM- and lubricant-specific trends over time. Pearson’s correlation coefficient (r) was used to evaluate linear relationships between average COF (COF) and testing variables of contact stress, sliding velocity, and average testing time (AvgTime). Positive r values indicated direct relationships, whereas negative r values indicated inverse relationships, with values closer to +1 or −1 reflecting stronger correlations. Corresponding *p*-values were reported to assess statistical significance.

#### 2.4.2. Wear Score Approach to Analysis

For each study, wear scores were independently assigned according to the rubric detailed in Table 3. When multiple criteria within a study were applicable to a given wear category, the higher value was used to represent that category. Scores from multiple observers were then averaged for subsequent analyses. Inter-rater reliability of wear scoring was assessed using the intraclass correlation coefficient (ICC). The two-way random-effects model for absolute agreement [ICC(2,1)] was chosen because all raters scored all samples, and the goal was to assess the reliability of absolute wear scores across raters rather than relative ranking. Finally, wear score distributions were visualized using violin plots to illustrate variability across studies.

COF values corresponding to each wear score were plotted, and linear regression models were fitted using ordinary least squares to evaluate the relationship between COF and wear. Regression outputs included the slope, intercept, 95% confidence intervals (CI), coefficient of determination (R^2^), and *p*-values for regression coefficients, which were reported to assess statistical significance. Mean wear scores and the standard error of the mean (SEM) were also calculated and reported. Pearson’s correlation coefficient (r) was used to evaluate linear relationships between wear score and COF and testing variables of contact stress, sliding velocity, and total sliding distance.

#### 2.4.3. Predictive Modeling

To evaluate the relationship between experimental variables and outcomes, linear regression models were fitted with either COF or wear score as the dependent variable, depending on the analysis. The full predictive model included all candidate predictors and was refined using backward elimination, sequentially removing the predictor with the highest *p*-value—that is, the variable contributing least to the model. For the COF model, the candidate predictors were contact stress, sliding velocity, and average testing time, whereas for the wear model, they included COF, contact stress, sliding velocity, and sliding distance. The model was then refitted, and *p*-values were recalculated for the remaining variables. This iterative process continued until all remaining predictors met a predefined significance threshold (commonly *p* < 0.05) or only the intercept remained. The goal was to simplify the model while maintaining explanatory power, reducing potential overfitting and multicollinearity. Statistical significance of individual predictors was assessed via t-tests on the regression coefficients (β). Model outputs included regression coefficients, R^2^, adjusted R^2^, root mean squared error (RMSE), and F-statistics, with corresponding *p*-values reported. Model fit was visually assessed by plotting actual versus predicted values, including an identity reference line (y = x). Model diagnostics were considered to ensure that results were broadly consistent with the assumptions of linear mixed-effects modeling.

## 3. Results

### 3.1. Results of Literature Search and Screening

**Figure 2.**
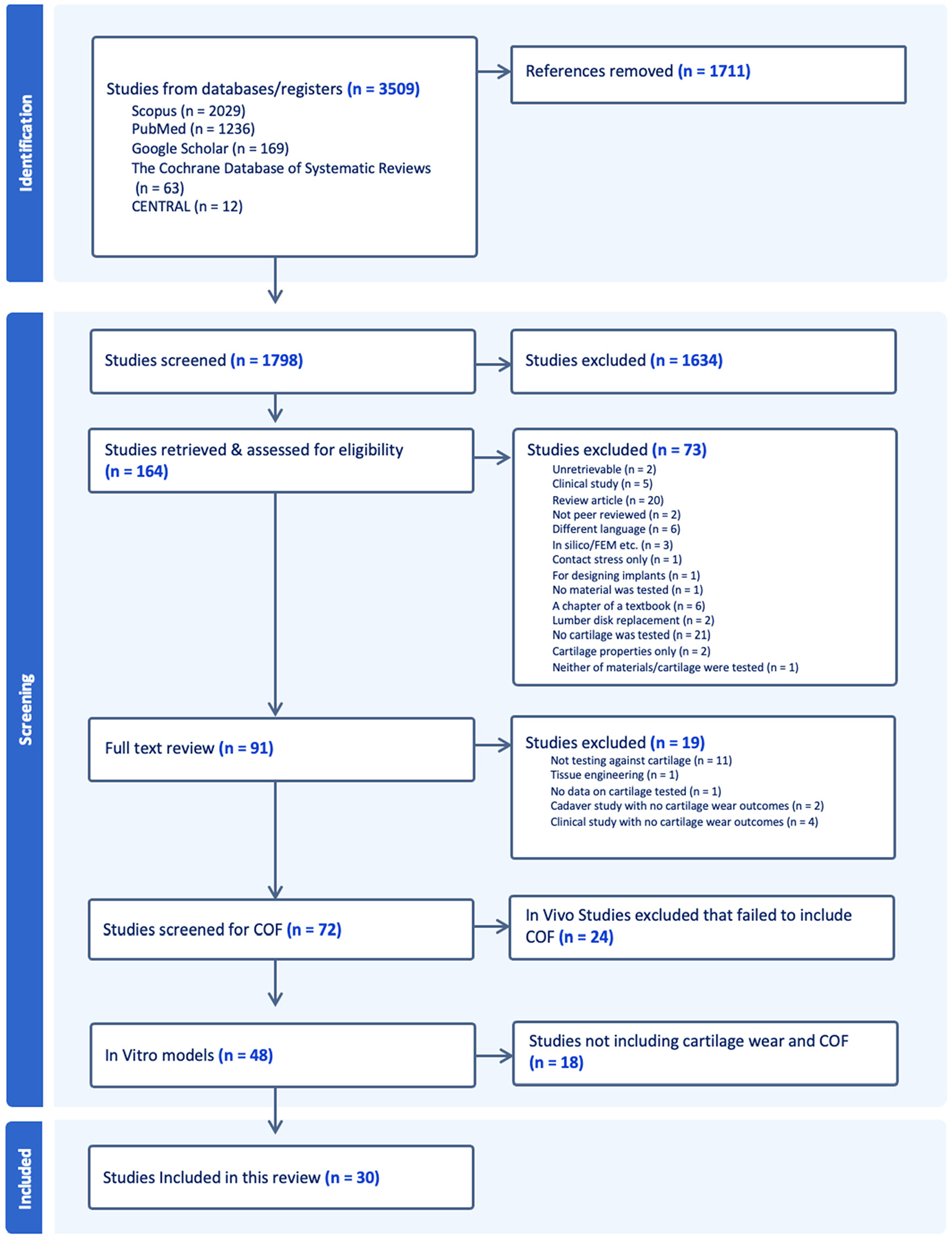
PRISMA flow diagram for study selection process for the review.

The initial search returned 3509 titles, from both clinical and basic science studies. After removing 1711 duplicates, 1798 titles and abstracts were screened according to predefined inclusion and exclusion criteria. Following full-text review, 30 [22–51] in vitro studies were included in the final analysis, each of which reported both a COF value and a corresponding measure of cartilage wear, as illustrated in Figure 2. Although our search strategy did not explicitly exclude in vivo studies, none were included in the final set, as none of them reported COF values and therefore failed to meet the core inclusion criteria.

### 3.2. Hemiarthroplasty Bearing Material and Lubricants

**Figure 3.**
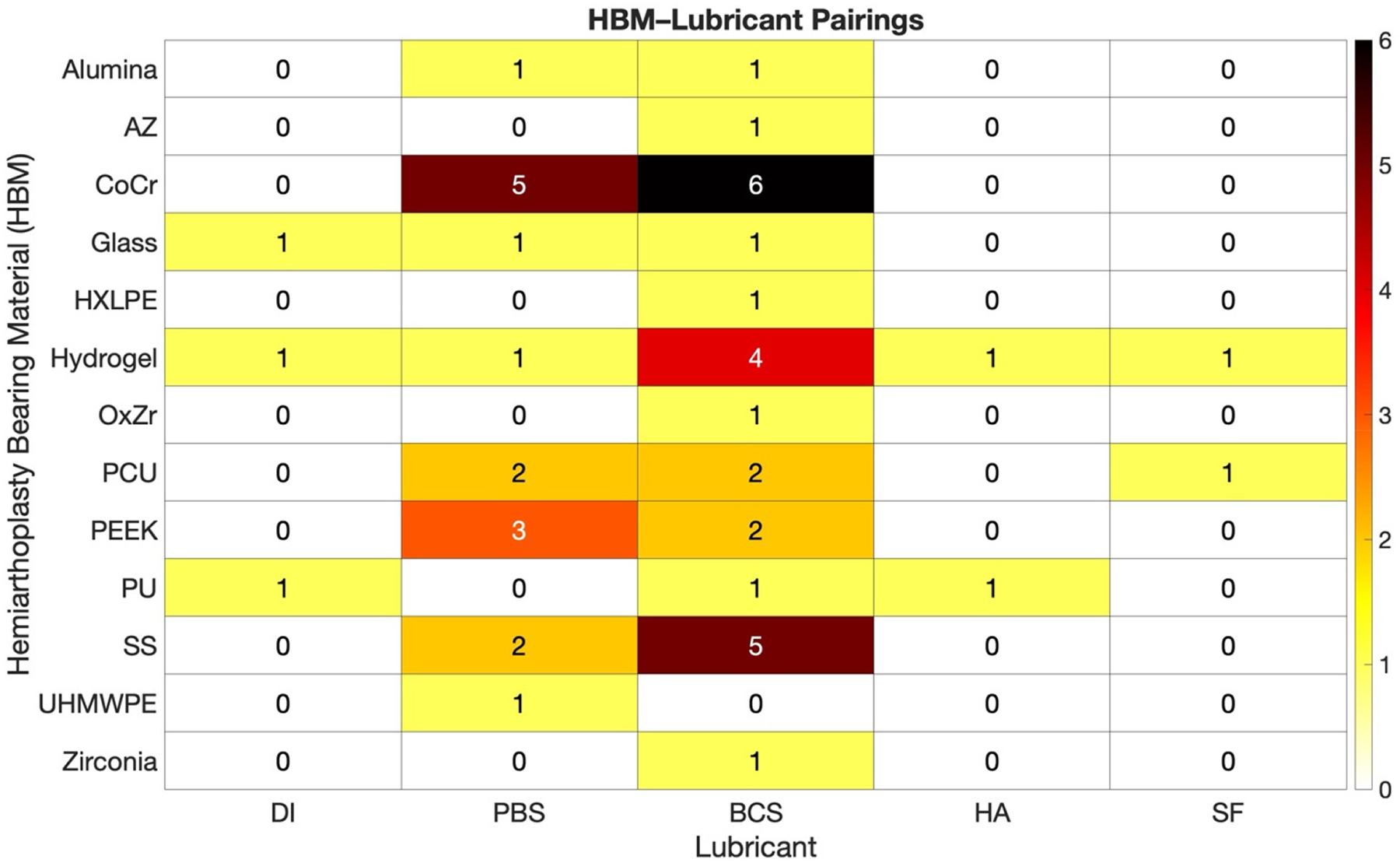
Heatmap of HBM–lubricant pairings extracted from 30 included in vitro studies. Each number within each cell represents the number of unique HBM–lubricant combinations tested, regardless of whether multiple combinations were reported within a single study. Darker colors indicate higher frequency. Abbreviations: AZ, Alumina-Zirconia; CoCr, Cobalt-Chromium Alloy; PCU, Polycarbonate-Urethane; SS, Stainless Steel; UHMWPE, Ultra-High Molecular Weight Polyethylene; PEEK, Polyether Ether Ketone; HXLPE, Highly Cross-Linked Polyethylene; PU, Polyurethane; OxZr, Oxidized Zirconium. DI, Deionized Water; PBS, Phosphate-Buffered Saline; Bovine Calf Serum; BCS, HA, Hyaluronic Acid; SF; Synovial Fluid.

Our review identified 13 different HBMs which were tested using 5 different lubricants. Figure 3 summarizes the number of studies that tested each unique combination of HBM and lubricant across the 30 included in vitro studies. Each cell represents the count of studies that reported a specific HBM–lubricant pairing, regardless of the number of experiments performed within a study. If a single study tested multiple HBMs or lubricants, it was counted once for each unique combination.

The most frequently studied pairings were cobalt-chromium (CoCr) in bovine calf serum lubricant (BCS; n = 6) and CoCr in phosphate-buffered saline (PBS; n = 5), followed by stainless steel (SS) in BCS (n = 5). Other lubricants included de-ionized water (DI), hyaluronan (HA) containing solutions, and synovial fluid (SF). Overall, BCS and PBS were the most frequently used lubricants, and CoCr, SS, hydrogels, and PEEK were among the most tested HBMs. In general, grouping decisions were based on the bulk composition of the HBM. Detailed tables with HBM and lubricants specifics may be found in Supplementary Material.

Factors such as surface finish, roughness, or other treatments were not considered, as including these would have made meaningful cross-study comparisons impractical due to the diversity of reporting and experimental conditions. Hydrogel formulations were analyzed as a single group—including PVA-based hydrogels, biphasic hydrogels, and other formulations (hydrogel 30%, 35%, etc.)—which does not account for potential differences in composition, structure, or properties among individual hydrogels. A similar approach was applied to lubricants, with attempts to group the most similar types; however, although BCS was commonly used, its composition varied between studies (e.g., different protein concentrations or ratios), which could influence outcomes. These grouping approaches were necessary to simplify comparisons across studies, as the large diversity of HBM and lubricant formulations would otherwise have precluded meaningful analysis.

### 3.3. Coefficient of Friction

The 30 included studies reported a wide range of experiment durations (5 to 1670 min; Table 4), making direct comparisons of COF challenging. To standardize inter-study comparisons, COF data were extracted at 600, 1800, and 3600 s (10, 30, and 60 min), representing early, intermediate, and late stages of testing for most studies. This selection was guided by two factors: first, the prior scoping review [17] indicated that a one-hour testing duration was most commonly used in similar in vitro setups; second, the majority of included studies had total testing durations that encompassed these time points (as shown by the median and mode in Table 4), ensuring that data extraction captured comparable stages across studies without exceeding individual study durations.

Additionally, Table 4 summarizes key experimental conditions across studies, highlighting the wide variation in testing parameters and providing context for interpreting COF measurements at standardized time points; when data were not explicitly reported, values were derived through calculations, with details available in the Supplementary Material.

#### 3.3.1. Linear Model Fit of COF

The 600, 1800 and 3600 s intervals were used to analyze how the COF changed over time. Specifically, 101 measurements were collected at 600 s, 109 at 1800 s, and 104 at 3600 s, for a total of 314 data points across all time points.

The coefficient of variation (CV) was calculated for each HBM at the three measured time points (600, 1800, and 3600 s) to quantify variability in COF. HXLPE and SS exhibited the lowest average variability, at 20.9% and 26.5%, respectively. HBMs with moderate variability included Alumina (50.0%), hydrogel (37.6%), PU (48.2%), and CoCr (49.4%). The highest variability was observed for PEEK (81.5%) and PCU (114.3%). Silicone, UHMWPE, and zirconia each had only a single observation, so variability could not be assessed. Detailed CV values for each HBM are provided in Appendix A Table A1, while Table 4 summarizes the range of testing parameters across studies to provide context for interpreting frictional variability.

**Figure 4.**
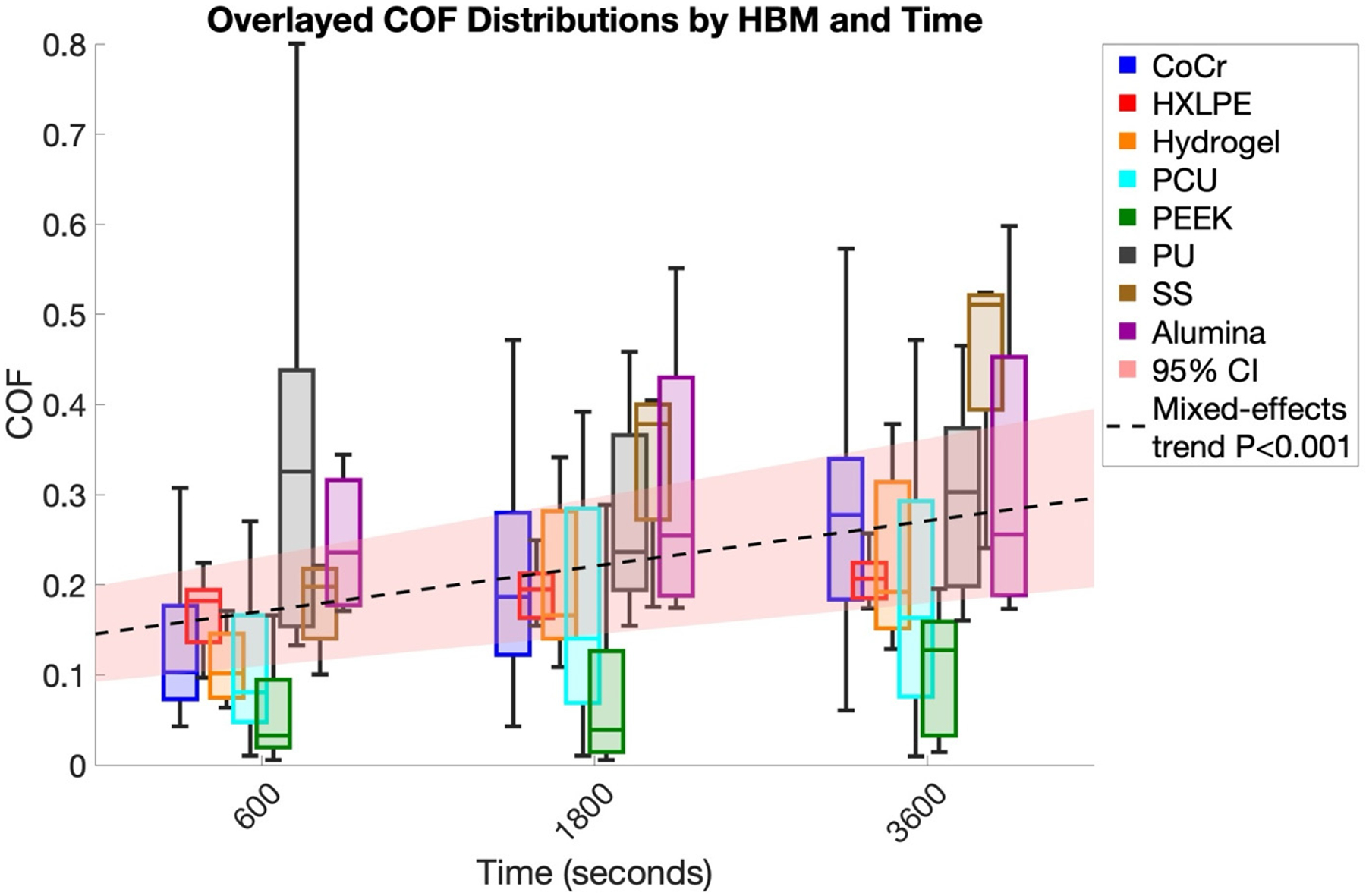
Box plots of COF at 600, 1800, and 3600 s across in vitro studies. The black dashed line depicts the predicted trend from a linear mixed-effects model (time as fixed effect, HBM as random effect), with the shaded area representing the 95% CI. The slope was 3.65 × 10^−5^ COF/s (95% CI: 2.45 × 10^−5^–4.84 × 10^−5^; *p* < 0.001), and the intercept was 0.127 (95% CI: 0.099–0.155), indicating a small but significant increase in COF over time. Only data points available at these specific time intervals were included; studies reporting only average COF values were excluded. For visualization purposes, each boxplot is grouped around its respective time point (600, 1800, or 3600 s) and slightly spaced horizontally to improve readability; this horizontal offset does not represent additional or intermediate time points. Abbreviations: CoCr, Cobalt-Chromium Alloy; PCU, Polycarbonate-Urethane; UHMWPE, Ultra-High Molecular Weight Polyethylene; PEEK, Polyether Ether Ketone; HXLPE, Highly Cross-Linked Polyethylene; PU, Polyurethane; SS, Stainless Steel.

To assess overall trends over time while accounting for variability across HBMs, a linear mixed-effects model was fitted with HBM as a random effect and time as a fixed effect. Across all 314 COF measurements, COF showed a small but statistically significant increase over time (Figure 4. Slope = 3.65 × 10^−5^ COF/s, 95% CI [2.45 × 10^−5^, 4.84 × 10^−5^], *p* < 0.001), with an intercept of 0.127 (95% CI [0.099, 0.155], R^2^ = 0.104). Pairwise comparisons between individual time points were not statistically significant.

To assess HBM- and lubricant-specific trends, linear models were fitted for each HBM-lubricant combination that had at least three measurements. Of the 20 combinations, 18 showed positive COF slopes over time, with 4 reaching statistical significance (CoCr–BCS and CoCr–PBS, Hydrogel–RS, SS–BCS), while 14 had positive but non-significant slopes. Two combinations exhibited non-significant negative slopes (see Appendix A, Table A2).

#### 3.3.2. Correlation of Testing Time, Contact Stress, and Velocity with COF

The correlation between average coefficient of friction (COF) and average of testing time (AvgTime), based on time points 600, 1800 and 3600s and final testing time for experiments that exceeded 3600s (Table 5), contact stress (Table 6), and velocity (Table 7) was assessed across multiple HBM–lubricant combinations. The correlation between COF and testing time (Table 5) was generally positive across HBM–lubricant combinations. Statistically significant increases in COF over time were found with CoCr–PBS and PCU–PBS.

For contact stress, statistically significant positive correlations (e.g., PCU–BCS, PEEK–BCS) suggest that as contact stress increases, COF also increases. Conversely, significant negative correlations (e.g., CoCr–BCS, Hydrogel–BCS) indicate an inverse relationship, where higher contact stress corresponds to lower COF. These mixed positive and negative correlations highlight that the direction of association may vary depending on the HBM–lubricant combination (Table 6).

Correlations between COF and velocity (Table 7) showed mixed results across HBM–lubricant pairs. Statistically significant negative correlations (e.g., CoCr–BCS, CoCr-PBS, Hydrogel–BCS) indicate COF decreases as velocity increases. While a significant positive correlation (PEEK–BCS and PCU-BCS) with the opposite trend of increasing COF with increasing contact stress.

#### 3.3.3. Predictive Modeling of COF

To better understand how input parameters influence friction behavior, linear regression models were developed using contact stress, velocity, and time as predictors. Analyses shown here are for CoCr. For other HBMs, either the available data were insufficient or the models did not converge successfully. Attempts to run these analyses for all HBMs are included in the Supplemental Material. This model aimed to predict the COF as a function of contact stress, velocity, and average time, using the general form:

(2) $$\text{COF}=\beta_{0}+\beta_{1}\times \text{Contact}\;\text{Stress}+\beta_{2}\times \text{Velocity}+\beta_{3}\times \text{AvgTime}+\epsilon$$

where COF is the coefficient of friction, β_0_ is the intercept, β_1_–β_3_ are the regression coefficients for each predictor (contact stress, velocity, and time), and ε is the error term. Separate linear regression models were constructed for CoCr under both BCS and PBS lubricant conditions. Linear regression models predicting COF under BCS–CoCr and PBS–CoCr conditions showed that time was a positive predictor of COF, reaching significance only in the PBS–CoCr model. In both models, sliding velocity was a significant negative predictor of COF, while Contact Stress was not significant (Table 8). Model fit statistics for the full model were higher for PBS–CoCr (adjusted R^2^ = 0.665) than for BCS–CoCr (adjusted R^2^ = 0.358) (Appendix A, Table A2).

**Figure A1.**
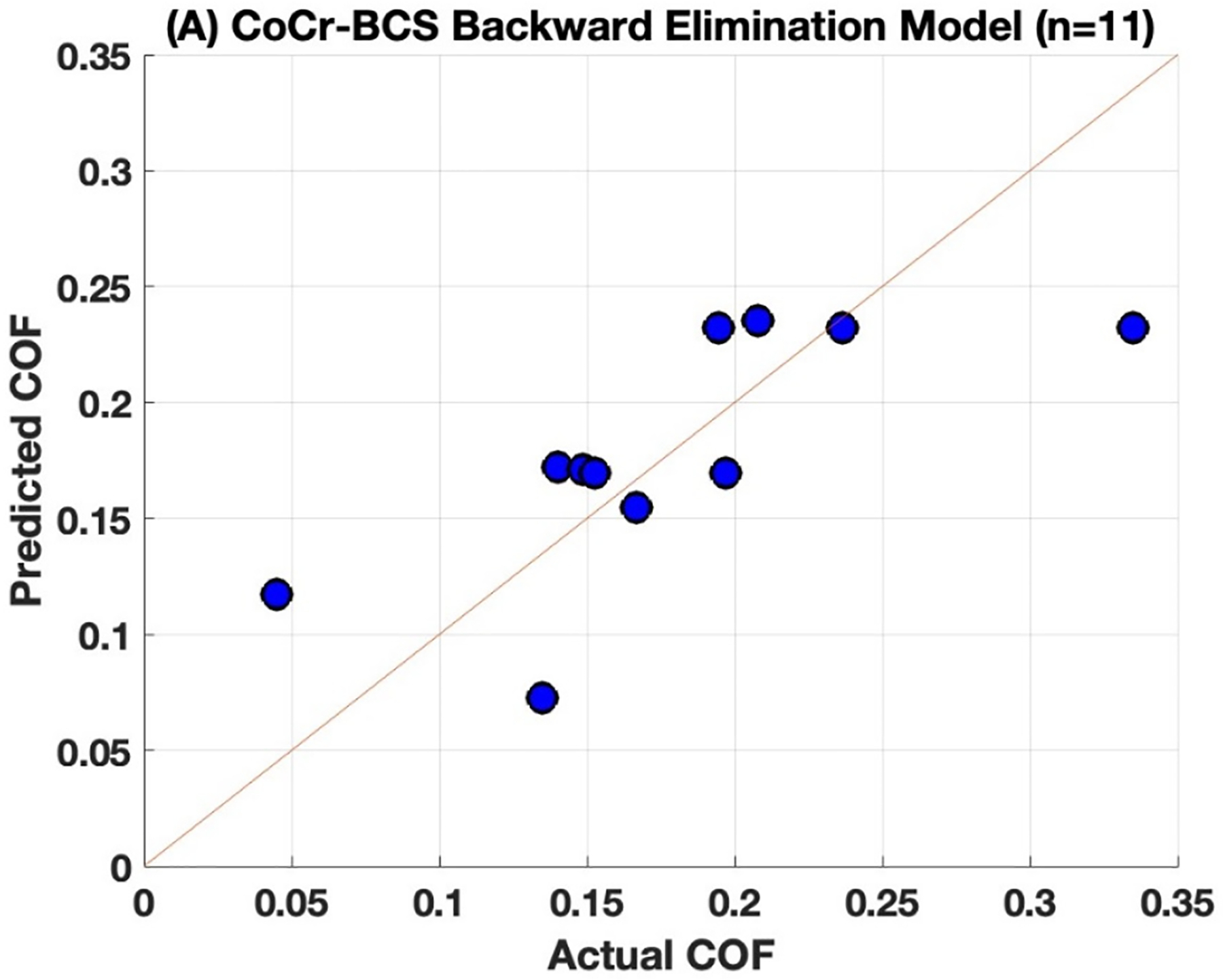

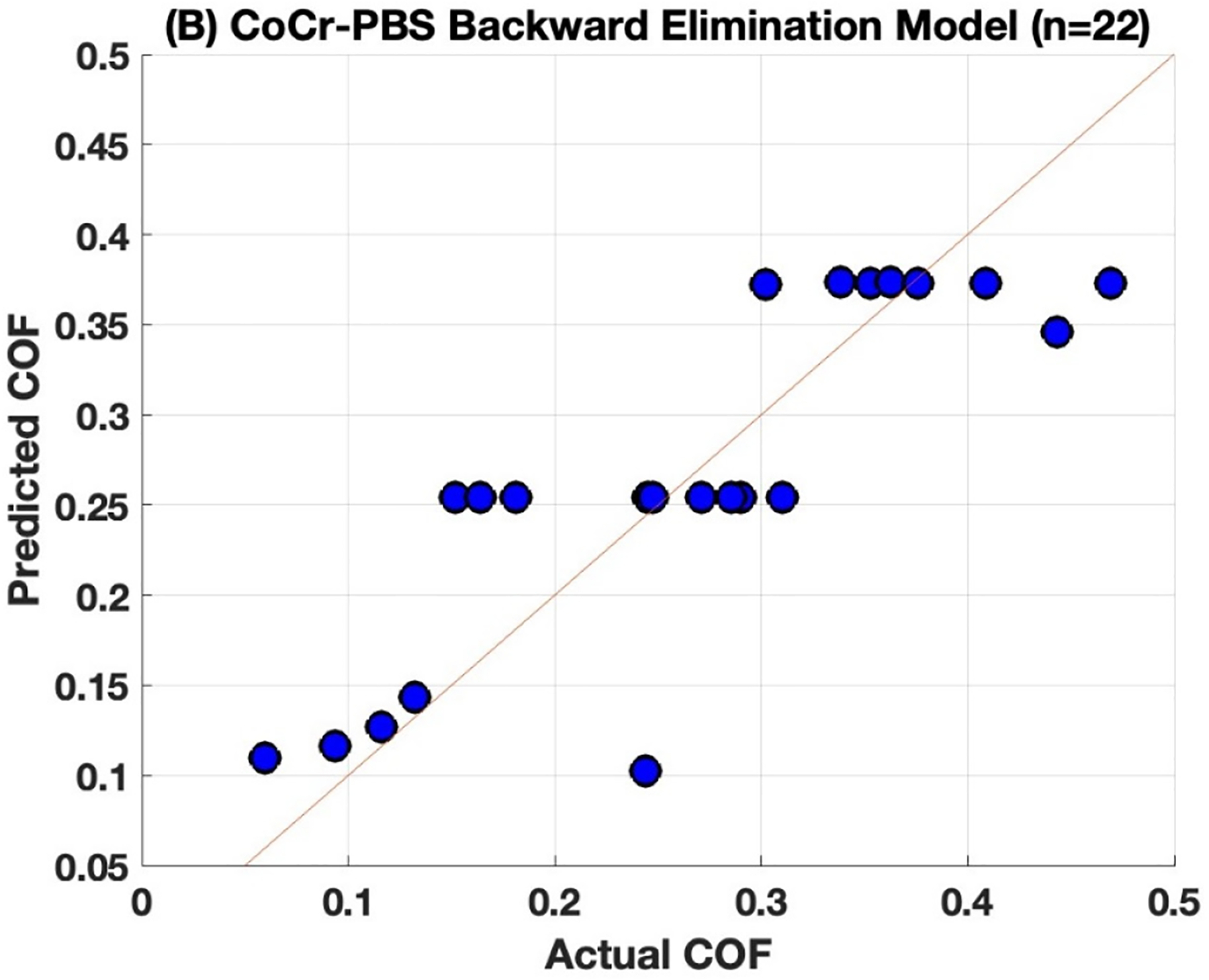
Residual plots for the predictive models of COF under BCS and PBS lubricant conditions after backward elimination. Blue circles represent individual residuals (observed minus predicted COF), and the orange line is a smoothed trend line illustrating any systematic deviation in the residuals across fitted values for (**A**) BCS–CoCr and (**B**) PBS–CoCr models.

After backward elimination, the BCS–CoCr model retained Contact Stress and AvgTime as the sole significant predictor of COF. In contrast, the PBS–CoCr model retained Sliding Velocity and AvgTime as predictors. For BCS–CoCr, higher contact stress was associated with lower COF and. The PBS–CoCr model indicated that time had a positive effect while sliding velocity showed a negative association (Table 9). Model fit statistics after elimination were higher for PBS–CoCr (adjusted R^2^ = 0.680) than for BCS–CoCr (adjusted R^2^ = 0.415) (Appendix A, Table A3 and Figure A1). These results suggest that the retained predictors explained a modest proportion of the variance in COF under each lubricant condition.

### 3.4. Wear Score Results

**Figure 5.**
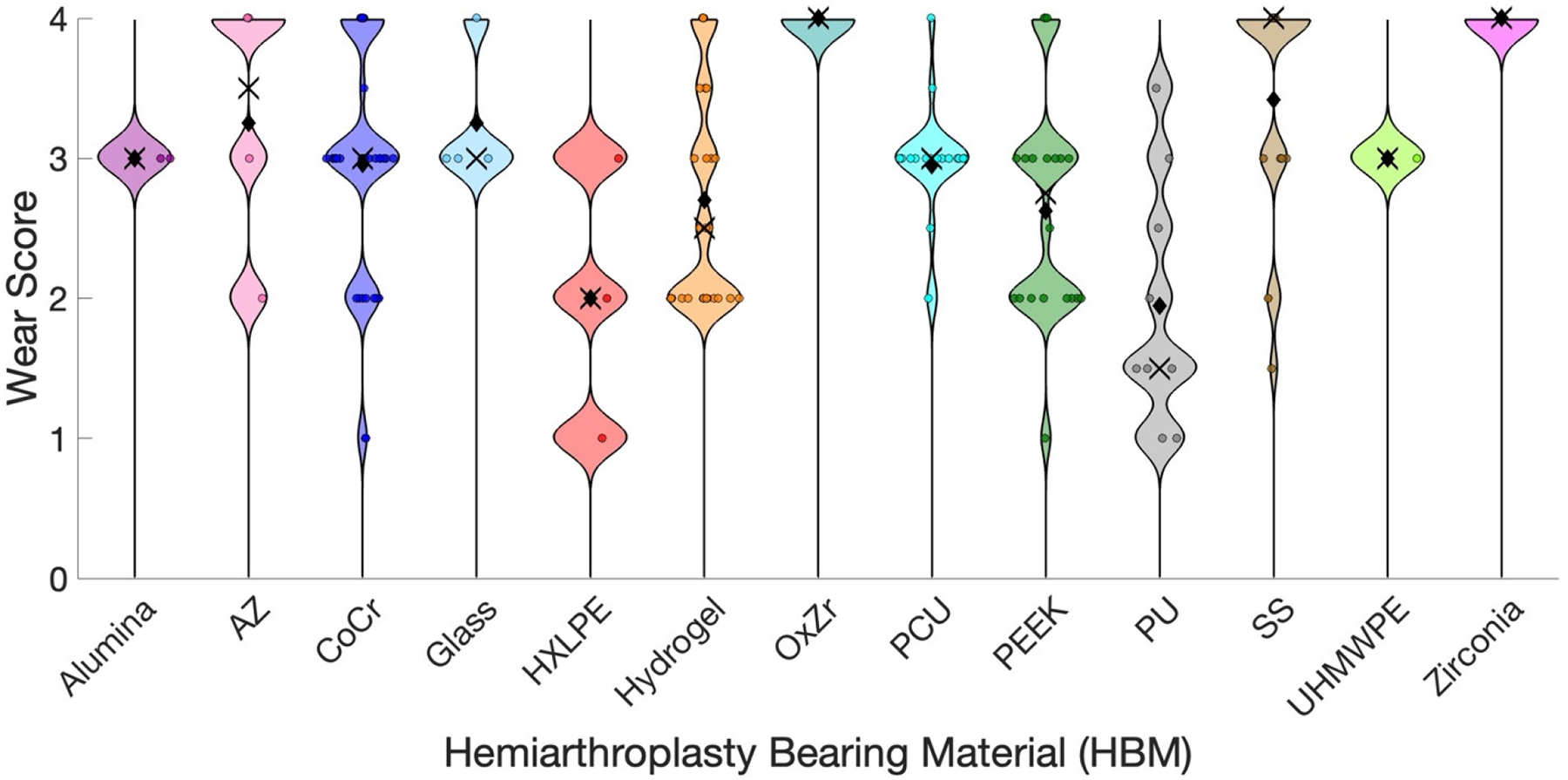
Wear Score Distribution for each HBM. Abbreviations: AZ, Alumina Zirconia composite; CoCr, Cobalt-Chromium Alloy; HXLPE, Highly Cross-Linked Polyethylene; OxZr, Oxidized Zirconium; PCU, Polycarbonate-Urethane; PEEK, Polyether Ether Ketone; PU, Polyurethane; SS, Stainless Steel; UHMWPE, Ultra-High Molecular Weight Polyethylene. Black diamonds indicate the mean and black ‘x’ symbols indicate the median. Each wear score is represented by a colored dot, with the color corresponding to the specific HBM (legend). The vertical position reflects the cartilage wear score (1 = low, 4 = high). Dot size or spread illustrates variability between wear score results.

Wear studies typically report outcomes once testing has concluded. In this review, cartilage wear was graded with defined criteria and then assigned a single semi-quantitative score, described in Table 3. Multiple independent raters (V.P.M., D.J.F., F.D.V., L.M.B., A.K.L.) assigned the semi-quantitative score. ICC values between raters ranged from 0.831 to 0.953 (mean ± SD: 0.891 ± 0.051), confirming strong interrater reliability. Figure 5 displays the distribution of wear scores by HBM, highlighting the variability in wear performance. The coefficient of variation (CV) of wear scores varied across HBMs, ranging from 0% (Alumina, OxZr) to 50% (HXLPE), with most HBMs having moderate variability (CV ≈ 15–31%) (Appendix B, Table A5).

#### Relationship Between Contact Stress, Velocity, Distance and Wear

Wear scores were further analyzed using Pearson’s correlation with contact stress (Table 10), sliding velocity (Table 11), and total sliding distance at the conclusion of testing (Table 12). Instead of using test duration directly, sliding distance was selected as the variable of interest because it more directly reflects the cumulative mechanical exposure of the cartilage–HBM interface. While duration captures only the length of time a test was run, sliding distance incorporates both time and velocity, thereby representing the actual extent of relative motion experienced by the tissue during testing.

Correlation analysis between wear and contact stress (Table 10) showed primarily positive but mostly non-significant associations across HBM–lubricant combinations. Notably, HXLPE–BCS and PCU–BCS demonstrated strong, statistically significant positive correlations, suggesting wear increased with contact stress in those cases.

Table 11 shows mixed correlations between wear and velocity across HBM–lubricant combinations. A statistically significant positive correlation was observed for CoCr–BCS, suggesting that wear increases with velocity in this pairing. Other combinations showed weak or non-significant correlations, with both positive and negative directions, indicating no consistent pattern across HBMs.

Table 12 shows that correlations between wear and sliding distance were generally weak and not statistically significant across HBM–lubricant combinations. Most combinations showed minimal association, and some even displayed slight negative correlations. Overall, no consistent relationship was observed.

### 3.5. Relationship Between COF and Wear

**Figure 6.**
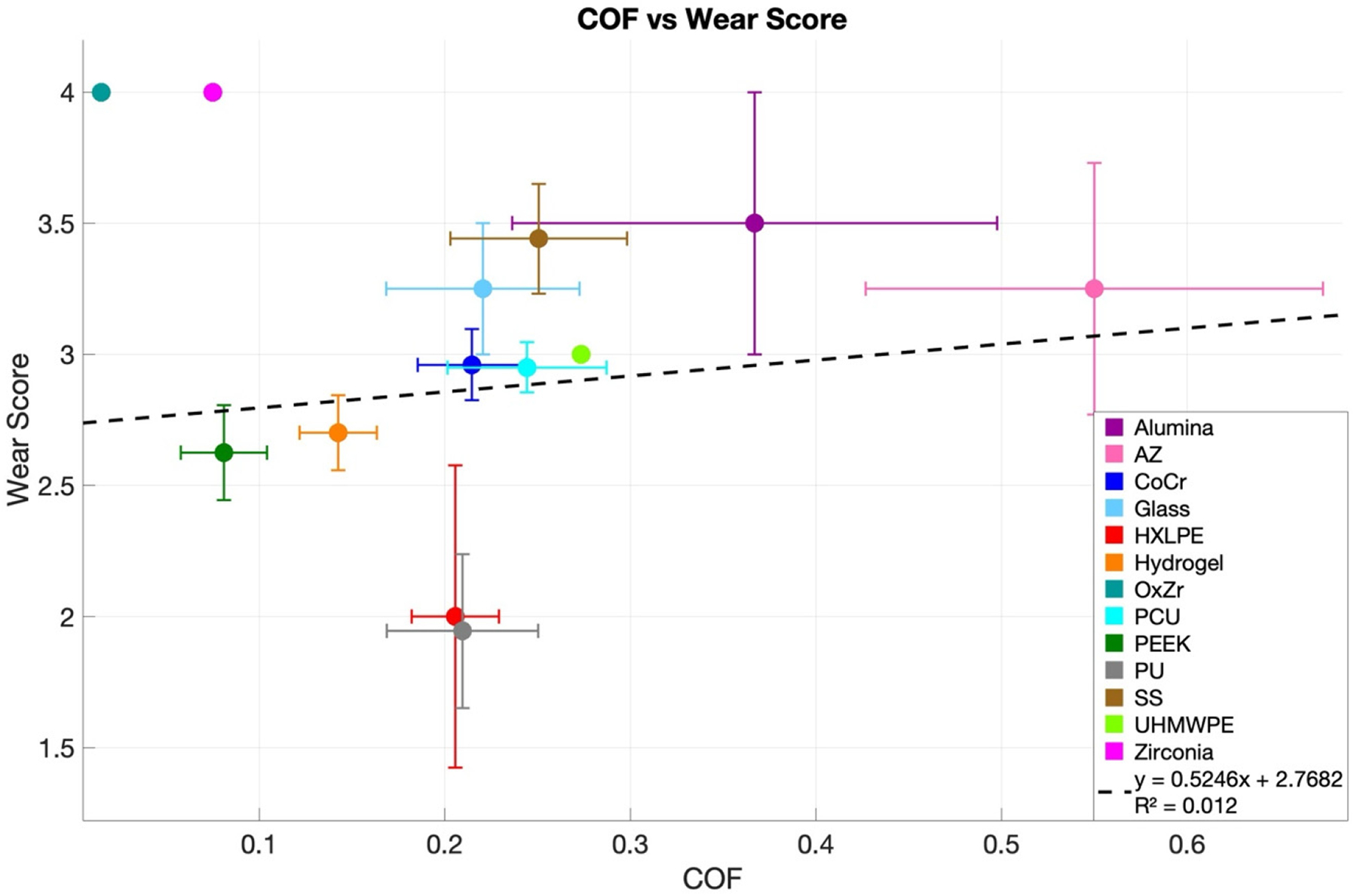
Mean COF and Wear scores (±SEM) for each HBM are shown. Individual data points used for the regression are displayed separately in Appendix A Figure A1. A linear regression fitted to individual data points across all HBMs is overlaid here (R^2^ = 0.012).

**Figure A2.**
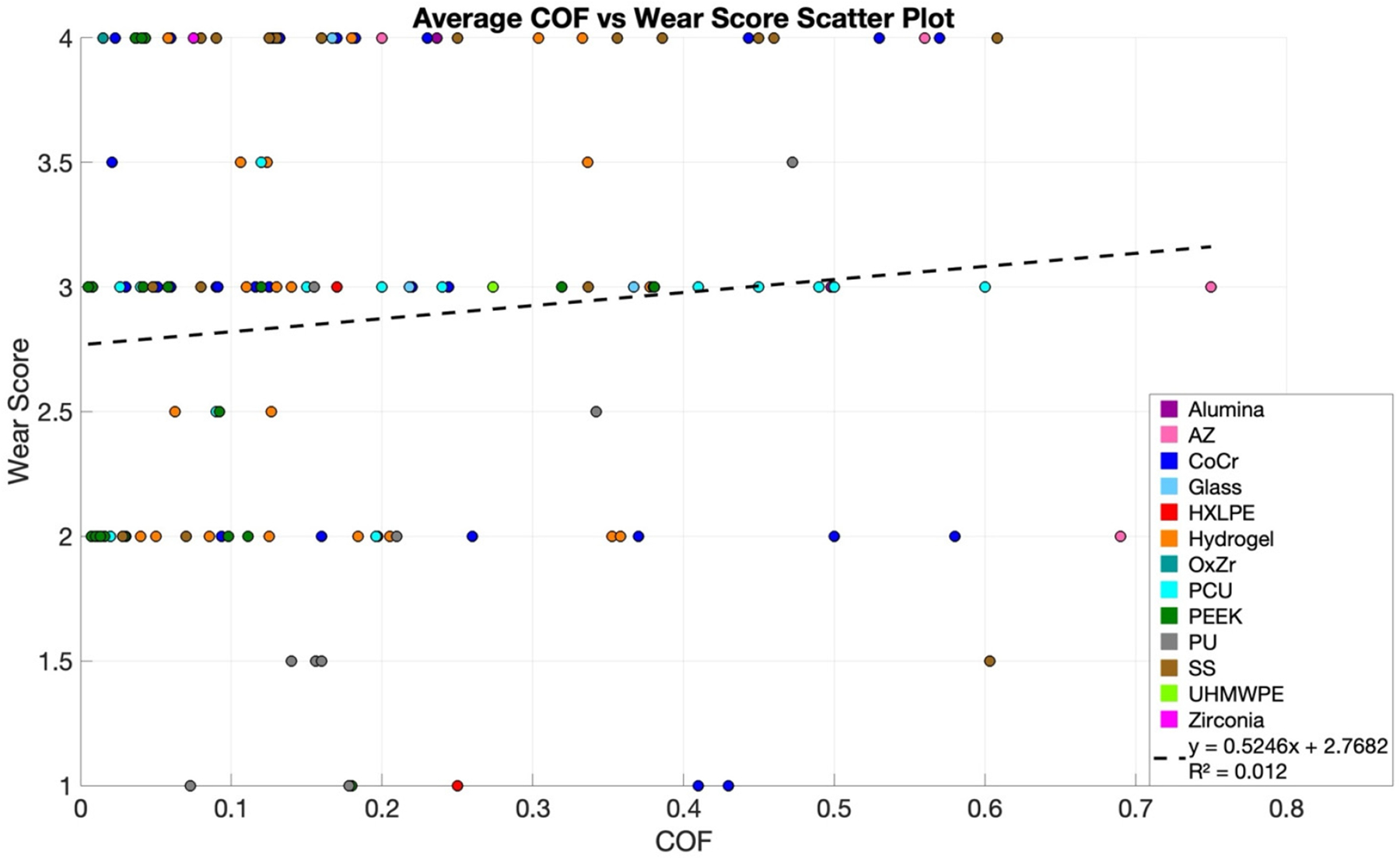
Average COF vs. Wear Score for HBMs Scatter plot. Linear fits derived from the dataset are overlaid in black dashed line.

COF and cartilage wear scores were collected and analyzed to evaluate the relationship between friction and cartilage degradation. Linear regression models were fitted to the data, with initial fits across all HBMs showing poor performance (R^2^ = 0.012) (Figure 6, Appendix B Figure A2).

Linear fit analyses of COF versus cartilage wear for each HBM and lubricant generally revealed weak relationships (Appendix B, Table A6). Most slopes were not statistically significant, and confidence intervals were wide, reflecting high variability and small sample sizes in several groups. Notable exceptions included PEEK in BCS lubricant, which exhibited a significant negative slope (slope = −14.8, 95% CI: −25.9 to −3.83, *p* = 0.014, n = 11), and PU in HA, which showed a significant positive slope (slope = 5.96, 95% CI: 3.23 to 8.69, *p* = 0.011, n = 4). Many HBMs tested in small groups (n ≤ 4) had slopes with very large confidence intervals or values effectively indistinguishable from zero.

#### 3.5.1. Correlation of COF and Wear Score

Pearson correlation analysis revealed mixed correlations between wear and average COF, with both positive and negative correlation coefficients observed, depending on the HBM–lubricant pairing (Table 13). Notably, PEEK–BCS showed a statistically significant negative correlation, whereas PU–HA showed a statistically significant positive correlation, indicating that the relationships occurred in opposite directions. Overall, the lack of a consistent trend indicates that the relationship between wear and COF is highly dependent on HBM and lubricant combinations.

#### 3.5.2. Predictive Modeling of Wear Score

Predictive modeling of wear was only possible with CoCr, as it provided the most complete dataset across all experimental conditions. A linear regression model was developed to evaluate the relationship between wear score and key tribological parameters. The model included coefficient of friction (COF), contact stress, sliding velocity, and sliding distance as predictors. While COF was included as a predictor in the model, its influence on wear is not yet fully established; it was incorporated to explore potential relationships. The model was constructed as:

(3) $$\text{WearScore}=\beta_{0}+\beta_{1}\times \text{COF}+\beta_{2}\times \text{ContactStress}+\beta_{3}\times \text{Velocity}+\beta_{4}\times \text{Distance}+\epsilon$$

where WearScore is assigned according to the scoring rubric (Table 3), β_0_ is the intercept, β_1_–β_4_ are the regression coefficients for each predictor (COF, contact stress, sliding velocity, and distance), and ε is the error term.

Separate linear regression models were constructed for CoCr under both BCS and PBS lubricant conditions (Table 14). The models predicting wear score showed poor fits, with adjusted R^2^ values of 0.093 (CoCr–BCS) and 0.095 (CoCr–PBS) (Appendix B, Table A8), reflecting the high variability in testing conditions and the limited dataset size. Following backward elimination, the CoCr–BCS model retained only sliding distance as a predictor, while the CoCr–PBS model retained only contact stress (Table 15). The models predicting wear score after backwards elimination slightly higher fits, but still poor with adjusted R^2^ values of 0.132 (CoCr–BCS) and 0.58 (CoCr–PBS) (Appendix B, Table A8). Importantly, COF was not a significant predictor in either model, indicating that friction alone did not reliably predict wear under these experimental conditions.

### 3.6. Risk of Bias Assessment Results

All 30 studies were independently scored using the QUIN tool [21] by three authors (VM, FDV, and LB). Based on the QUIN cutoffs, 27 studies were classified as low risk of bias (>70%) and 3 studies were classified as medium risk (50–70%). No studies met the criteria for high risk of bias (<50%) (Appendix C, Table A9). Any scoring discrepancies were discussed among the reviewers, and a consensus was reached

## 4. Discussion

This systematic review evaluated 30 studies to investigate the relationships between coefficient of friction (COF), wear, and key testing parameters—contact stress, sliding velocity, sliding distance, and testing time—across various hemiarthroplasty bearing materials (HBM) and lubricants. Overall, the findings indicate that COF demonstrates limited and inconsistent association with cartilage wear and therefore should not be considered a reliable standalone surrogate outcome. Although friction generally increased over time, reflecting the biphasic properties of cartilage, these changes were not reliably predictive of damage [52]. The effects of contact stress and sliding velocity on COF and wear varied depending on the specific HBM–lubricant combinations. Regression analyses identified sliding velocity and time as significant predictors of COF, whereas wear was more closely linked to contact stress and cumulative sliding distance in select cases. These findings underscore not only the complex, HBM-dependent nature of tribological performance in hemiarthroplasty contexts, but also suggest that the duration of typical experiments may have been too short to yield meaningful wear results. Nevertheless, understanding factors that drive friction and wear is essential for optimizing HBM design and improving the longevity and functionality of hemiarthroplasty implants.

The variability in COF reported across HBMs provides insight into both HBM behavior and experimental sensitivity. HXLPE and SS exhibited low variability (CVs of 20.9% and 26.5%, respectively), suggesting stable, reproducible frictional performance; however, this apparent stability may be influenced by smaller sample sizes (15 for HXLPE, 26 for SS) compared with CoCr, which had 84 observations and moderate variability (49.4%). In contrast, PEEK and PCU showed very high variability in COF, which likely reflects a combination of inherent material properties and inconsistencies in experimental test setups. Factors such as contact stress, sliding velocity, lubrication conditions, and other subtle differences between studies may have amplified this variability, as summarized in Table 4. Materials with moderate variability, including Alumina, hydrogel, PU, and CoCr, indicate partial stability but some susceptibility to experimental conditions. High variability implies that frictional performance is less predictable, and care must be taken when interpreting results or comparing materials, as apparent differences in COF could arise from experimental fluctuations rather than intrinsic material properties. For HBMs with only a single observation (silicone, UHMWPE, zirconia), variability could not be assessed, highlighting the need for additional measurements to evaluate consistency.

Taken together, these findings highlight the complexity of tribological behavior in hemiarthroplasty bearing materials, with COF and wear influenced by both material properties and experimental conditions. To systematically address these issues, this review focused on three core questions: (1) which factors influence the coefficient of friction (COF) in hemiarthroplasty bearings, (2) which factors determine cartilage wear, and (3) how COF and wear are related to each other. Accordingly, the discussion is organized into Sections 4.1–4.3, which examine each of these questions in detail.

### 4.1. Determinants of Coefficient of Friction

In this review, COF exhibited a statistically significant increase over time across all 314 measurements with a slope of 0.131 units/h (Figure 4). The mixed-effects model detected a consistent upward trend, indicating that friction progressively rose during testing. The low R^2^ value (0.104) suggests that time explains only a small fraction of the total variability, highlighting the influence of other factors such as HBM type, surface properties, lubricant, or experimental contact conditions (e.g., stationary vs. migrating cartilage contact). While pairwise comparisons between specific time points were not significant, the overall trend captured by the mixed-effects analysis underscores subtle temporal effects that may be missed in simpler analyses. Measurements beyond the 95% confidence interval (outliers) likely reflect HBM-specific variability or deviations in local test conditions. Furthermore, the model indicates that HBM- and lubricant-specific responses dominate the observed variation. Most HBM–lubricant combinations showed positive slopes (Appendix A, Table A2), though few reached statistical significance, emphasizing that frictional behavior is strongly dependent on the specific HBM pairing, experimental conditions (e.g., velocity, contact stress, lubricant), and the time intervals (600, 1800, and 3600 s) over which this review examined the data. These findings highlight the heterogeneity inherent in tribological interactions and suggest that simple time-dependent models are insufficient to capture the full complexity of HBM–cartilage behavior.

The above findings with testing duration are consistent with prior tribological studies involving cartilage and cartilage-analogous materials, where frictional behavior is known to evolve with prolonged loading [52–54]. Time-dependent increases in COF have been repeatedly reported in cartilage tribology, especially under stationary or repeated loading conditions. This phenomenon is largely attributed to the exudation of interstitial fluid from the cartilage matrix, which reduces the fluid load support and increases the role of the solid matrix in load bearing [53,55–57]. Forster and Fisher et al. demonstrated that, under static loading, COF values of bovine cartilage increased a COF of approximately 0.01 at 5s to 0.3 at 45 min, highlighting the role of fluid depressurization [55]. Similar trends have been observed in biphasic cartilage models, where friction increases as the pressurized fluid phase diminishes over time and the load is transferred to the porous solid matrix [56,58]. Ateshian et al. further showed that contact migration plays a critical role: under migrating contact, interstitial fluid pressure is maintained, and friction remains low; however, under stationary or repeated contact, fluid exudation dominates, and COF progressively rises [53,56]. This is consistent with the significant time effects seen across several tested HBM–lubricant pairs. Although cartilage lubrication is inherently nonlinear and biphasic, nonlinear or biphasic COF–time models were not applied due to heterogeneous reporting and limited availability of continuous time-resolved COF data across studies.

The effect of contact stress on the coefficient of friction (COF) varied among HBM–lubricant pairings (Table 6). Statistically significant positive correlations were observed for PEEK–BCS and PCU–BCS, suggesting that as contact stress increases, COF also rises. In contrast, significant negative correlations were observed for CoCr–BCS, HXLPE–BCS, and Hydrogel–BCS. The effect of contact stress on soft materials is complex. Higher stress flattens asperities, which increases the real contact area, and in turn, may increase adhesion. This may lead to a higher COF. On the other hand, a larger contact area may enhance fluid retention at the interface, thereby lowering COF [59]. Caution is advised for interpreting the strong negative correlation for HXLPE, which is based on only five data points. Collectively, these patterns underscore that the stress–COF relationship is HBM and lubricant-specific, with both deformation and lubrication mechanisms contributing, and that limited sample sizes constrain the strength of inference.

Similarly, the effect of sliding velocity on COF was variable and exhibited HBM-specific trends (Table 7). PEEK–BCS and PCU–BCS showed positive correlations, indicating that higher velocity increased friction. These counterintuitive trends can be explained by transitions in lubrication regime and the mechanical compliance of the material. Soft and compliant materials deform at both the surface and bulk levels, which reduces effective fluid-film thickness and prevents a monotonic decrease in COF with speed [60,61]. The interplay between pressure and material deformation may lead to a film thickness, which does not monotonically increase with speed [62]. Other mechanisms, like lubricant entrainment and retention may play a role, too. It has been reported that PEEK, a hard polymer, may promote lubricant removal faster than its replenishment, leading to higher friction [63]. PCU, a soft and compliant polymer, also showed a positive trend but based on only five data points, warranting cautious interpretation. In contrast to the above, CoCr under both BCS (*n* = 11) and PBS (*n* = 22) lubrication exhibited a negative correlation between sliding velocity and COF, consistent with rigid surfaces supporting mixed or fluid-film lubrication, where higher velocity enhances fluid entrainment and film thickness. Unlike compliant polymers, CoCr does not deform appreciably under load, so the shape of the Stribeck curve is primarily governed by the soft cartilage counterface and its ability to support elastohydrodynamic lubrication (EHL). Together, these findings highlight that the influence of sliding velocity on COF is strongly dependent on HBM compliance, surface mechanics, and lubricant film stability, with limited sample sizes constraining confidence for some pairings.

Predictive models were developed to examine the influence of contact stress, sliding velocity, and average testing time on the average COF of CoCr BCS and PBS lubricated conditions. The differences in frictional behavior between BCS and PBS can be partly explained by their distinct lubricating properties. BCS has a higher viscosity than water, which likely facilitates the onset of EHL even at relatively low sliding velocities [58,64–69]. For both CoCr-BCS and CoCr-PBS, time was positively associated with COF (Table 8) indicating that friction increases over the course of testing as biphasic lubrication effects diminish, as previously discussed. The full models explained some portion of the variance in COF, with adjusted R^2^ values of 0.665 for CoCr–PBS (n = 22) and 0.359 for CoCr–BCS (n = 11). The higher R^2^ for PBS likely reflects the larger number of observations, while the lower value for BCS may be influenced by its limited sample size (Appendix A, Table A2).

To identify the most influential factors affecting COF, backward elimination was applied to the full models (Table 9). In both models, time was retained and positively associated with CoCr–BCS (β = 0.004, *p* = 0.047) and CoCr-PBS (β = 1.65 × 10^−4^, *p* = 0.001), again highlighting the importance of testing time in COF measurements. In the reduced CoCr–BCS model, contact stress was an additional predictor of COF (β = −0.083, *p* = 0.017), while in CoCr–PBS, sliding velocity (β = −0.026, *p* < 0.001) was significant. The reduced models showed moderate explanatory power, with adjusted R^2^ values of 0.415 for BCS–CoCr and 0.680 for PBS–CoCr (Appendix A, Table A3; Figure A1). Overall, while these predictors capture some of the variation in COF, additional factors likely contribute to the observed variability.

Although this review did not assess the impact of surface roughness, it is well established that friction and wear generally increase with greater roughness. However, reporting of surface roughness in the literature is often incomplete as it is typically limited to the average roughness (Ra), which does not capture important spatial characteristics, such as whether peaks are sharp or valleys are rounded. Future studies should provide more detailed descriptions of surface features to clarify their influence on friction and wear.

### 4.2. Determinants of Wear

Wear was reported using various outcome measures across the included studies. To enable cross-study comparison, a standardized scoring system was developed (Table 3), incorporating commonly reported wear metrics. No consensus currently exists within the field regarding the optimal method for assessing cartilage wear, further contributing to variability in reported outcomes [17]. These standardized wear scores were then paired with corresponding COF values for statistical analysis. The model demonstrated high intraclass correlation coefficients (ICCs), indicating strong internal consistency, although external validation has not been performed. The distribution of HBM wear scores (Figure 5) and CV analysis (Appendix B, Table A5) highlights that some HBMs, such as Alumina, OxZr, and UHMWPE, exhibited no variability in wear, although this is likely influenced by very small sample sizes (n ≤ 2) and may not reflect true material behavior. HBMs like HXLPE and PU showed high variability, suggesting less predictable wear behavior, whereas CoCr, Hydrogel, PCU, and SS demonstrated moderate variability with sufficient sample sizes, indicating more consistent performance. These differences may reflect intrinsic HBM properties, testing conditions, or both, and emphasize the importance of considering variability alongside mean wear when comparing HBM performance. Subsequently, the wear scores were analyzed in relation to input variables—contact stress, velocity, and sliding distance—as well as COF, to explore potential relationships to cartilage wear.

Pearson correlation analysis demonstrated variable relationships between wear and contact stress, velocity, and sliding distance across HBM–lubricant combinations (Tables 10–12). Significant positive associations between wear and contact stress were observed for CoCr–PBS, HXLPE–BCS, and PCU–BCS, indicating that higher contact stress was associated with increased wear in these pairs of HBM-lubricant. Other combinations generally showed positive but non-significant correlations. Correlations with velocity were inconsistent, ranging from weakly positive to negative, and none reached statistical significance. Associations between wear and sliding distance were also weak and pre-dominantly non-significant across all pairs, with only CoCr–BCS showing a borderline significant positive correlation.

The variable and generally weak correlations between wear and biomechanical parameters may, at least in part, reflect the limited durations and sliding distances of the test. In the studies reviewed, testing times ranged from 5 min to 28 h, representing only a small fraction of the approximately 1389 h (58 days) required to complete a standard ISO 14242-1 [70] hip simulator wear test, which evaluates total hip prosthesis wear under physiologic loading and motion conditions, or the many thousands of hours of expected wear in clinical use. Similarly, the sliding distances observed in these experiments, averaging 64.2 m and reaching a maximum of 9 km, were substantially lower than those expected under standardized hip or knee simulator protocols. To illustrate the magnitude of this difference, Saikko’s force track analysis provides a useful reference for estimating the sliding distance achieved in a full ISO hip simulator test [71]. Based on this analysis, the track length under load in an ISO hip simulator is approximately 1.58 × r, corresponding to 25.28 mm for a 32 mm head. Over 5 million cycles, this results in a total sliding distance of approximately 126 km. Collectively, these observations highlight that the relatively short-duration testing reported in the studies included in our review may not be sufficient to capture the cumulative effects of contact stress, sliding velocity, and total sliding distance on wear [65].

### 4.3. Relationship of COF and Wear

**Figure 7.**
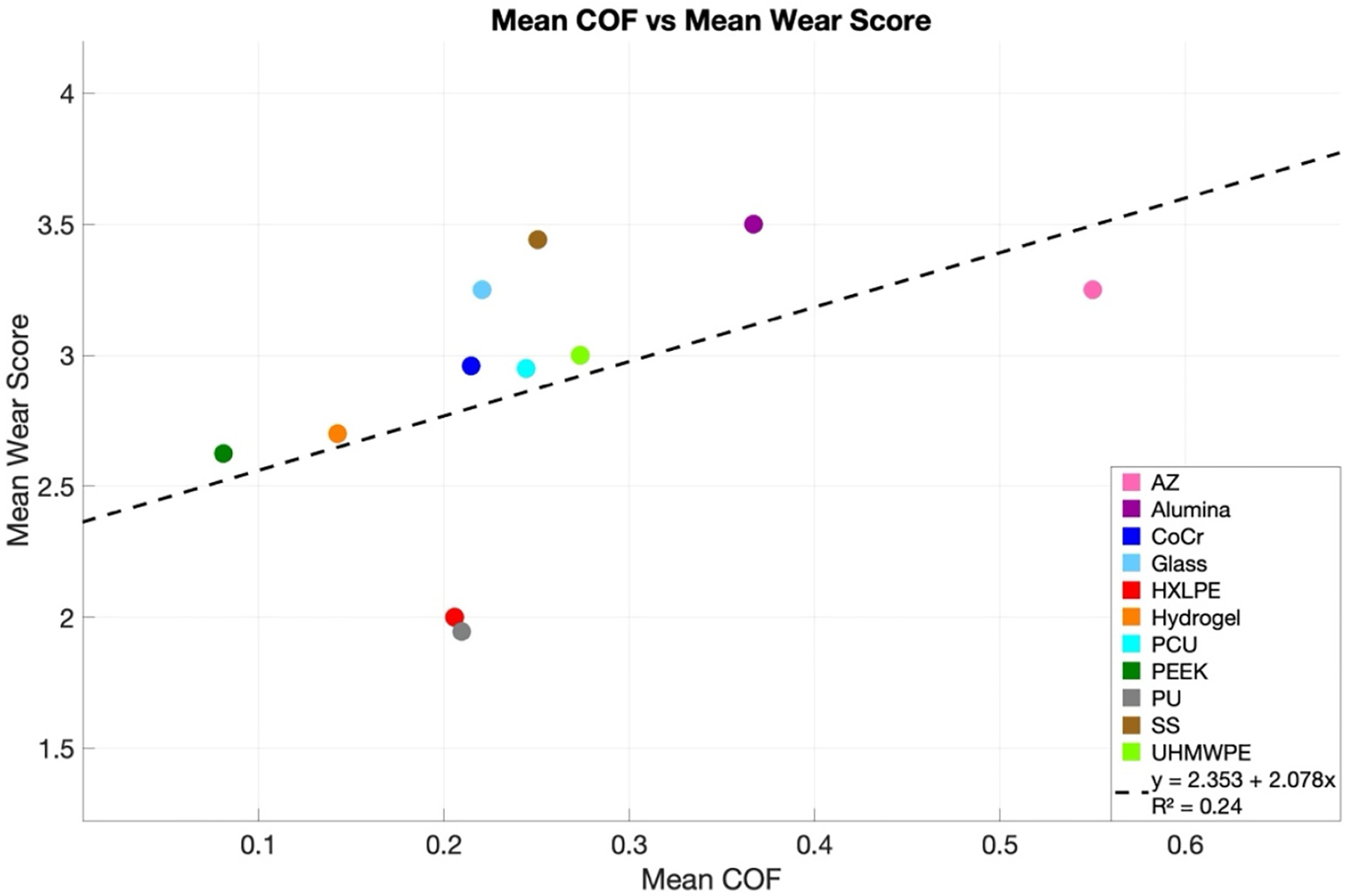
Mean coefficient of friction (COF) versus mean wear score for each hemiarthroplasty bearing material. Each colored point represents the HBM-specific average COF and wear score across all observations, with colors corresponding to materials as indicated in the legend. OxZr and Zirconia were excluded from this plot. The dashed line shows a linear regression fit to the mean values (R^2^ = 0.24), highlighting a modest association between COF and wear after data aggregation. This plot demonstrates that, while raw COF measurements are highly variable, trends linking friction to cartilage wear may emerge when data are averaged by HBM.

**Figure A4.**
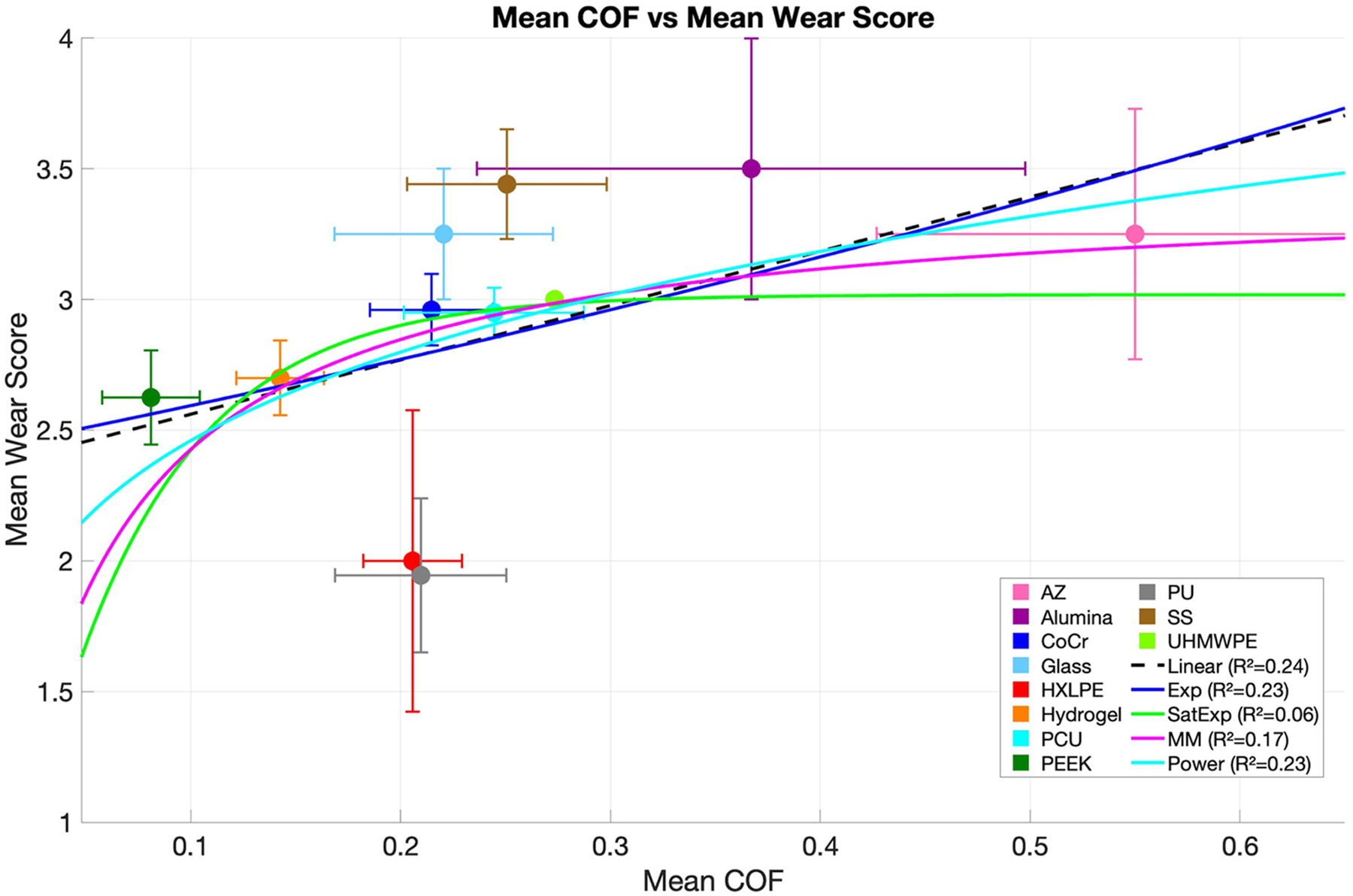
Relationship between mean coefficient of friction (COF) and mean wear score across hemiarthroplasty bearing materials (HBMs), with outliers removed. Each point represents the mean COF and mean wear score for a specific HBM–lubricant combination. A linear regression was applied to the averaged data, showing a modest correlation (R^2^ = 0.24). Alternative models—including exponential, saturating exponential, Michaelis-Menten, and power-law fits—were also evaluated, but the linear model provided the best fit (highest R^2^) and the most interpretable relationship. This suggests that underlying trends in COF versus wear become more apparent after aggregation, as averaging reduces variability from individual measurements.

To address the third core question of the review, how COF and cartilage wear are related, Section 4.3 evaluates their association across HBMs and lubricants, showing that COF alone is generally not a reliable predictor of wear. A linear regression model applied to COF measurements versus wear scores—included every available data point from all HBMs—showed a poor fit (R^2^ = 0.01) (Figure 6; Appendix B, Figure A2). This result indicates that COF alone explains very little variability in wear. Examination of individual HBM–lubricant combinations yielded similar findings: most showed flat or inconsistent slopes with wide confidence intervals, reflecting substantial variability in wear outcomes (Appendix B, Table A7). Interestingly, when COF and wear values were averaged (with outliers removed), a modest association emerged (R^2^ = 0.24) (Figure 7). This suggests that underlying trends may become visible after data aggregation, since averaging attenuates data fluctuations. Alternative models were explored, including exponential, saturating exponential, Michaelis-Menten, and power-law fits, but the linear regression yielded the highest R^2^ (Appendix D, Figure A4). As shown in Table 4, the experimental input parameters varied widely across experiments, with contact stress ranging from 0.1 to 31.3 MPa, velocity from 0.5 to 100 mm/s, and test durations spanning 5 to 1670 min. Sliding distance and cycle counts likewise covered several orders of magnitude. Such heterogeneity in test conditions likely contributes to the inconsistent COF–wear relationships observed, as both friction and wear are highly sensitive to loading, kinematics, and test duration. The lack of standardized protocols complicates cross-study comparisons and underscores the need for community-defined benchmarks for friction testing and reproducible measures of wear in hemiarthroplasty research.

Taken together, these findings demonstrate that COF alone is not a reliable predictor of cartilage wear across heterogeneous HBMs. Nonetheless, it is possible that COF does influence wear, but current testing approaches—confounded by widely varying input parameters—do not capture this relationship adequately. Moving forward, the field would benefit from coordinated efforts to (1) define standardized friction testing protocols and (2) establish consistent, validated wear outcome measures applicable to hemiarthroplasty studies.

## 5. Limitations

Overall, the methodological quality of the included studies was acceptable, with most demonstrating low risk of bias according to the QUIN tool. Notably, none of the included studies reported blinding of outcome assessment, which was the QUIN criterion most consistently underreported and represents an area for improvement in future tribological research. One limitation of this review was the lack of detailed, time-resolved data for velocity or contact stress, unlike what was available for different time points and COF. Consequently, Pearson correlations and predictive modeling of COF were performed using the average COF and time across 600, 1800, 3600 s, and the final reported time point to obtain a single mean COF and mean time for each experiment, which were then used in analyses with contact stress and velocity. This approach limits the analysis because it removes temporal resolution and variability, potentially masking nonlinear trends, time-dependent effects, and peaks or plateaus in COF, which could affect the accuracy of correlations and predictive models.

Another limitation is the absence of an objective, universally accepted quantitative metric for wear. To enable cross-study comparisons, a custom scoring system was developed using commonly reported wear indicators. While this approach introduces some subjectivity, score assignment was consistent among evaluators, as evidenced by high intraclass correlation coefficients (ICCs); however, the method has not been independently validated.

Data were extracted at the level of individual experimental configurations rather than by study. Consequently, studies reporting multiple configurations contributed more heavily to the analysis, while datasets with as few as three points were still included. This may have introduced an imbalance in study influence. Additional limitations include the lack of detailed information for some HBMs on surface finish or other treatments, limited data availability for several HBMs (particularly 8 of 13) and for certain lubricants, specifically DI, HA, and SF, and variability in testing methods and experimental setups, which could influence outcomes. As highlighted in Table 4, these studies exhibited large variability in key testing parameters such as contact stress, velocity, frequency, and duration, further contributing to differences in measured outcomes across studies.

Additional limitations relate to HBM grouping and heterogeneity. Hydrogels were grouped together in this review because each study often used unique, study-specific adaptations of hydrogel materials, such as differences in composition, crosslinking, or processing. While this allowed for broader comparison, it may obscure material-specific effects on tribological behavior.

Further limitation of this review is that quantitative cartilage wear rates could not be evaluated or compared across studies. None of the included studies reported time-normalized, volumetric, or mass-loss–based cartilage wear metrics. Instead, cartilage damage was assessed using qualitative or semi-quantitative indicators such as surface fibrillation, histological changes, or structural deformation. As a result, wear rate estimation and lifetime predictions could not be performed, and comparisons were limited to relative wear severity rather than absolute wear kinetics.

Finally, it is important to distinguish between variability arising from biological tissue properties and variability resulting from experimental design. While some differences in outcomes may reflect inherent heterogeneity in cartilage samples or HBM properties, a substantial portion of variability is likely attributable to differences in experimental parameters, such as contact stress, sliding velocity, lubricant type, and testing duration. This distinction should be considered when interpreting correlations and predictive models, as variability from these sources may differently impact study findings.

## 6. Conclusions

This systematic review highlights the complex relationship between cartilage friction and wear in hemiarthroplasty. Thirty in vitro studies examining COF, cartilage wear, and key testing parameters, including contact stress, sliding velocity, sliding distance, and test duration, were evaluated across various hemiarthroplasty bearing materials and lubricants. While COF generally increased over time, reflecting cartilage’s biphasic behavior, its association with actual wear was inconsistent and highly dependent on the specific material and lubricant combination. Wear outcomes were more closely linked to contact stress and cumulative sliding distance in some cases, whereas sliding velocity and test duration primarily influenced COF. Collectively, these findings demonstrate that COF alone is not a reliable predictor of cartilage wear, underscoring the need to consider the testing setup, methodological variations, and other biomechanical factors when evaluating cartilage performance in hemiarthroplasty.

## Supplementary Material

List of Suppl Material

Suppl Material 1

Suppl Material 2

Suppl Material 3

Suppl Material 4

Suppl Material 5

**Supplementary Materials:** The following supporting information can be downloaded at: https://www.mdpi.com/article/10.3390/lubricants14010018/s1.

## Figures and Tables

**Table 1.** Criteria for Inclusion and Exclusion in the Literature Search.

Inclusion Criteria
Evaluations of articular cartilage in contact with HBMs, including mechanical testing on benchtop models and in vivo studies
Pre-clinical and clinical studies assessing cartilage wear from articulation with HBMs, including X-ray imaging (such as joint space narrowing or bony erosion)
Exclusion Criteria
Studies focused solely on modeling or FEA
Clinical assessments that rely only on PROMs (e.g., KOOS, WOMAC, SF-12)
Studies published in languages other than English. Review Articles, Textbook chapters
Studies that did not include both cartilage wear and COF/friction measurements

Finite Element Analysis (FEA), Patient-Reported Outcome Measures (PROM), Knee Injury and Osteoarthritis Outcome Score (KOOS), Western Ontario and McMaster Universities Osteoarthritis Index (WOMAC), and the 12-Item Short Form Health Survey (SF-12).

**Table 2.** Data for Evaluating Hemiarthroplasty Bearing Materials (HBM) and Cartilage Interaction.

Category	Variables (with Units Where Applicable)
HBM	Type (metal, ceramic, polymer), Sub-types (e.g., CoCr, SS, HXLPE, PU, etc.)
Inputs	Contact stress (MPa), Sliding velocity (mm/s), Test duration (s), Sliding distance (m), Lubricant type (e.g., BCS, PBS, HA, RS)
Outputs—COF	COF at 600, 1800, 3600 s; Steady-state COF; Average COF; Other reported values
Outputs—Cartilage Wear	Quantitative (e.g., surface roughness, thickness changes), Qualitative (e.g., surface damage)

**Table 3.** Scoring system for cartilage damage based on relative performance (0–4 scale). Higher scores indicate more severe cartilage wear and deformation across multiple assessment modalities.

Score	Cartilage Wear	Macroscopic Appearance	Microscopy Surface Damage	Histology	Surface Roughness (Ra/Rz Change)	Recovery (Strain/Compression)	Surface Deformation	Relative Category
0	No wear	Smooth, glossy, unchanged	No damage	Normal matrix & cells	No change (baseline)	Full elastic recovery	No deformation	None
1	≤25th percentile	Slight dulling, intact	Microabrasions or faint scratches	Mild surface fibrillation	<20% change	Slight recovery loss	Mild deformation	Lowest group
2	>25th–≤50th percentile	Wear paths, shallow pits	Surface abrasion or subsurface cracks	Mid-zone damage, PG loss	20–50% change	Incomplete recovery	Moderate deformation	Mild group
3	>50th–≤75th percentile	Deep cracks, delamination	Extensive fissures, breakdown & delamination	Deep damage, necrosis	>50% change, irregular	Major residual deformation	Severe deformation	Moderate group
4	>75th percentile	Extensive delamination, fragmentation	Severe breakdown	Severe matrix loss & necrosis	Extreme change	Permanent deformation	Catastrophic deformation	Severe group

**Table 4.** Summary of testing parameters across all studies. Values represent descriptive statistics for contact stress, sliding velocity, testing frequency, duration, distance, and number of cycles. The table includes mean, median, mode, standard deviation (SD), minimum, and maximum values, illustrating the range and variability of experimental conditions used in the included studies. When inputs were not explicitly provided, they were derived through calculations to estimate missing data; details are provided in the Supplementary Material.

Parameter	Contact Stress (MPa)	Velocity (mm/s)	Frequency (Hz)	Testing Duration (min)	Distance (m)	Cycles (n)
Mean	4.09	15.0	0.695	294	401.7	15,504.8
Median	0.905	10	0.5	86.7	64.2	3600
Mode	0.5	4.0	1.0	60.0	14.4	3600
SD	7.08	19.5	0.545	45.0	1398.2	36,434.4
Minimum	0.1	0.5	0.005	5.0	1.8	9.0
Maximum	31.3	100	2.27	1670.0	9000.0	204,624

**Table 5.** Pearson correlation coefficients (r) and *p*-values for the relationship of time and COF. Boldface indicates significant findings.

HBM	Lubricant	Pearson r	*p*-Value	Observations (n)
Alumina	BCS	0.781	0.429	3
CoCr	BCS	0.103	0.762	11
**CoCr**	**PBS**	**0.649**	**0.001**	**22**
HXLPE	BCS	−0.401	0.504	5
Hydrogel	BCS	0.373	0.140	17
Hydrogel	RS	0.094	0.824	8
**PCU**	**BCS**	**0.901**	**0.037**	**5**
PCU	HA	0.965	0.169	3
PCU	PBS	0.097	0.855	6
PEEK	BCS	−0.175	0.549	14
PEEK	PBS	0.039	0.9408	6
PU	HA	0.204	0.523	12
SS	BCS	0.392	0.262	10

**Table 6.** Pearson correlation coefficients (r) and *p*-values for the relationship contact stress and COF. Boldface indicates significant findings.

HBM	Lubricant	Pearson r	*p*-Value	Observations (n)
CoCr	BCS	−0.458	0.157	11
CoCr	PBS	−0.210	0.349	22
HXLPE	BCS	−0.928	0.023	5
**Hydrogel**	**BCS**	*−* **0.839**	**0.002**	**17**
Hydrogel	RS	0.300	0.470	8
**PCU**	**BCS**	**0.901**	**0.037**	**5**
PCU	HA	−1.94 × 10^−16^	1	3
PCU	PBS	−0.395	0.439	6
PEEK	BCS	0.317	0.270	14
SS	BCS	−0.352	0.318	10

**Table 7.** Pearson correlation coefficients (r) and *p*-values for the relationship velocity and COF. Boldface indicates significant findings.

HBM	Lubricant	Pearson r	*p*-Value	Observations (n)
CoCr	BCS	−0.136	0.690	10
**CoCr**	**PBS**	*−* **0.670**	**<0.001**	**22**
**Hydrogel**	**BCS**	*−* **0.982**	**<0.001**	**10**
Hydrogel	RS	−0.208	0.620	8
**PCU**	**BCS**	**0.901**	**0.037**	**5**
**PEEK**	**BCS**	**0.925**	**<0.001**	**14**
SS	BCS	−0.352	0.317	10

**Table 8.** Estimated regression coefficients, standard errors, t-statistics, and *p*-values for the linear regression models predicting COF with Contact Stress, Velocity, and Time as predictors for BCS–CoCr and PBS–CoCr conditions. Boldface indicates significant findings.

Model	Coefficient (β)	Estimate	Standard Error	t-Statistic	*p*-Value
BCS–CoCr COF	Intercept (β_0_)	0.106	0.061	1.73	0.126
	Sliding Velocity (β_1_)	−0.003	0.002	1.62	0.149
	**Contact Stress (β**_**2**_ **)**	**−0.089**	**0.030**	**0.030**	**0.023**
	AvgTime (β_3_)	0.003	0.011	0.011	0.061
PBS–CoCr COF	Intercept (β_0_)	0.253	0.041	8.55	<0.001
	**Sliding Velocity (β**_**1**_ **)**	**−0.027**	**0.004**	**−4.33**	**<0.001**
	Contact Stress (β_2_)	0.002	0.006	0.434	0.670
	**AvgTime (β**_**3**_ **)**	**1.69 × 10** ^**−4**^	**0.006**	**3.74**	**<0.001**

**Table 9.** Estimated regression coefficients, standard errors, t-statistics, and *p*-values for the linear regression models predicting coefficient of friction (COF). After backward elimination, the BCS–CoCr model retained Velocity as the sole predictor, while the PBS–CoCr model retained both Time and Velocity as predictors. Boldface indicates significant findings.

Model	Coefficient	Estimate	Standard Error	t-Statistic	*p*-Value
BCS–CoCr COF	Intercept (β_0_)	0.111	0.058	1.93	0.090
	**Contact Stress (β**_**1**_ **)**	−**0.002**	**0.028**	−**2.98**	**0.047**
	**AvgTime (β**_**3**_ **)**	**0.004**	**0.002**	**2.35**	**0.017**
PBS–CoCr COF	Intercept (β_0_)	0.265	0.039	6.62	<0.001
	**Sliding Velocity (β**_**2**_ **)**	−**0.013**	**0.007**	−**2.04**	**<0.001**
	**AvgTime (β**_**3**_ **)**	**1.65 × 10** ^**−4**^	**<0.001**	**3.81**	**0.001**

**Table 10.** Pearson correlation coefficients (r) and *p*-values for Wear and Contact Stress. Boldface indicates significant findings.

HBM	Lubricant	Pearson r	*p*-Value	Observations (n)
AZ	BCS	0.64	0.357	4
CoCr	BCS	0.355	0.135	19
CoCr	PBS	0.45	0.052	19
Hydrogel	BCS	0.06	0.833	14
Hydrogel	RS	1.57 × 10^−16^	1	3
**HXLPE**	**BCS**	**1**	**<0.001**	**3**
**PCU**	**BCS**	**0.95**	**<0.001**	**9**
PEEK	BCS	0.29	0.382	11
PEEK	PBS	−0.1	0.798	9
PU	BCS	0.99	0.098	3
SS	BCS	0.32	0.248	15

**Table 11.** Pearson correlation coefficients (r) and *p*-values for Wear and Velocity. Boldface indicates significant findings.

HBM	Lubricant	Pearson r	*p*-Value	Observations (n)
CoCr	BCS	0.11	0.654	19
CoCr	PBS	0.07	0.761	19
Hydrogel	BCS	−0.26	0.368	14
**PCU**	**BCS**	**1.0**	**<0.001**	**9**
PEEK	BCS	−0.50	0.114	11
SS	BCS	0.30	0.270	15

**Table 12.** Pearson correlation coefficients (r) and *p*-values for Wear and Distance.

HBM	Lubricant	Pearson r	*p*-Value	Observations (n)
AZ	BCS	0.64	0.364	4
CoCr	BCS	0.42	0.07	19
CoCr	PBS	−0.06	0.813	18
Hydrogel	BCS	0.13	0.654	14
PCU	BCS	0.23	0.545	9
PEEK	BCS	−0.08	0.814	11
SS	BCS	0.21	0.462	15

**Table 13.** Pearson correlation coefficients (r) and *p*-values for the relationship Wear and COF.

HBM	Lubricant	Pearson r	*p*-Value	Observations (n)
AZ	BCS	−0.68	0.322	4
CoCr	BCS	0.08	0.760	19
CoCr	PBS	−0.09	0.726	19
HXLPE	BCS	−0.98	0.117	3
Hydrogel	BCS	1.9 × 10^−3^	0.995	14
Hydrogel	RS	−0.06	0.964	4
Hydrogel	SF	0.85	0.152	4
PCU	BCS	0.28	0.468	9
PCU	PBS	0.06	0.881	9
PEEK	BCS	−0.71	0.014	9
PEEK	PBS	0.58	0.099	9
PU	BCS	0.28	0.821	3
PU	HA	0.99	0.011	4

**Table 14.** Estimated regression coefficients, standard errors, t-statistics, and *p*-values for the linear regression models predicting Wear Score with COF, Contact Stress, Velocity, and Distance as predictors for CoCr–BCS and CoCr–PBS conditions.

Model	Coefficient (β)	Estimate	Standard Error	t-Statistic	*p*-Value
CoCr–BCS Wear Score	Intercept (β_0_)	1.94	0.696	2.78	0.014
	COF (β_1_)	0.386	2.72	0.142	0.889
	Contact Stress (β_2_)	0.162	0.251	0.646	0.529
	Velocity (β_3_)	−0.004	0.012	−0.319	0.754
	Distance (β_4_)	0.0001	<0.0001	1.25	0.233
CoCr–PBS Wear Score	Intercept (β_0_)	2.19	0.888	2.47	0.028
	COF (β_1_)	−1.64	2.45	−0.670	0.514
	Contact Stress (β_2_)	0.255	0.109	2.34	0.036
	Velocity (β_3_)	−0.245	0.168	−1.46	0.169
	Distance (β_4_)	0.002	0.002	1.09	0.297

**Table 15.** Estimated regression coefficients, standard errors, t-statistics, and *p*-values for the linear regression models predicting coefficient of friction (COF). After backward elimination, the CoCr–BCS model retained distance as a predictor, while the CoCr–PBS model retained contact stress as a predictor.

Model	Coefficient (β)	Estimate	Standard Error	t-Statistic	*p*-Value
CoCr–BCS Wear Score	Intercept (β_0_)	1.93	0.136	14.1	<0.001
	Distance (β_4_)	<0.001	<0.001	1.93	0.071
CoCr–PBS Wear Score	Intercept (β_0_)	1.43	0.312	4.60	<0.001
	Contact Stress (β_2_)	0.140	0.067	2.09	0.052

**Figure A3.**
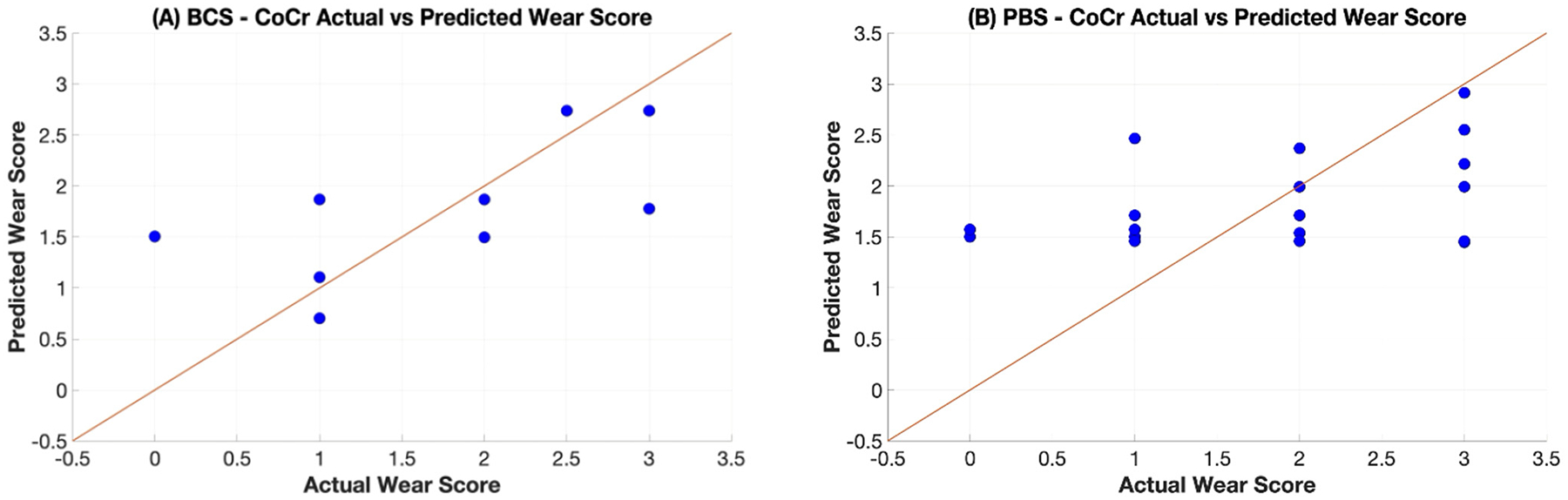
Residual plots for the predictive models of COF under BCS and PBS lubricant conditions after backward elimination. Blue circles represent individual residuals (observed minus predicted COF), and the orange line is a smoothed trend line illustrating any systematic deviation in the residuals across fitted values for (**A**) BCS–CoCr and (**B**) PBS–CoCr models.

## Data Availability

The original data presented in the study is available in the Supplementary Material.

## Appendix A

**Table A1.** Coefficient of variation (CV, %) of the coefficient of friction (COF) for each HBM at 600, 1800, and 3600 s, and the average CV across time points and respective count (n) of number of data points available. High CV values indicate greater variability in frictional behavior. HBMs with only single-study data (Silicone, UHMWPE, Zirconia) are included for reference, but variability could not be determined.

HBM	CV600 (%)	CV1800 (%)	CV3600 (%)	Average CV (%)	Observations (n)
Alumina	33.843	55.608	60.4	49.95	12
CoCr	56.971	51.844	39.316	49.377	84
HXLPE	28.343	19.122	15.214	20.893	15
Hydrogel	34.636	39.54	38.575	37.584	51
PCU	74.104	79.567	90.677	81.449	42
PEEK	123.67	121.11	98.097	114.29	53
PU	61.256	43.251	40.083	48.196	23
SS	30.913	24.687	24.01	26.537	26
Silicone	NaN	NaN	NaN	NaN	3
UHMWPE	NaN	NaN	NaN	NaN	3
Zirconia	NaN	NaN	NaN	NaN	2

**Table A2.** Results of linear regression models for the coefficient of friction (COF) versus time across HBM—lubricant combinations. Slopes are scaled to units of 10^−5^ COF/s and reported with 95% confidence intervals (CI), count (n), and significance. Bold entries indicate statistically significant positive trends (*p* < 0.05).

HBM	Lubricant	Slope (×10^−5^)	Lower 95% CI (×10^−5^)	Upper 95% CI (×10^−5^)	*p*-Value	Observations (n)
Alumina	BCS	0.45	−3.0	3.9	0.8	9
Alumina	PBS	8.2	0.45	16	0.29	3
**CoCr**	**BCS**	**2.7**	**0.62**	**4.8**	**0.017**	**33**
**CoCr**	**PBS**	**5.3**	**3.1**	**7.5**	**<0.001**	**66**
HXLPE	BCS	1.3	−0.26	2.8	0.13	15
Hydrogel	BCS	2.4	−0.19	5.1	0.08	39
**Hydrogel**	**RS**	**2.8**	**1.7**	**3.8**	**<0.001**	**21**
PCU	BCS	5.6	0.48	11	0.051	15
PCU	HA	−0.13	−0.71	0.45	0.67	9
PCU	PBS	2.5	−1.5	6.4	0.24	18
PEEK	BCS	1.2	−0.32	2.8	0.13	42
PEEK	PBS	2.7	−6.7	12	0.58	18
PU	HA	−1.9	−8.5	4.7	0.58	36
PU	RS	5.5	2.9	8.1	0.15	3
**SS**	**BCS**	**8.5**	**5.2**	**12**	**<0.001**	**30**
SS	RS	2.9	1.8	4	0.12	3
Silicone	RS	6.4	4.8	8	0.081	3
UHMWPE	PBS	8	5.3	11	0.11	3

**Table A3.** Model fit statistics including sample size (n), coefficient of determination (R^2^), adjusted R^2^, root mean squared error (RMSE), F-statistic, and overall model *p*-value for the linear regression models predicting average COF in BCS–CoCr and PBS–CoCr conditions.

Model	Observations (n)	R^2^	Adjusted R^2^	RMSE	F-Statistic	Model *p*-Value
CoCr–BCS COF	11	0.550	0.358	0.046	2.857	0.114
CoCr–PBS COF	22	0.713	0.665	0.060	14.9	<0.001

**Table A4.** Model fit statistics including sample size (n), coefficient of determination (R^2^), adjusted R^2^, root mean squared error (RMSE), F-statistic, and overall model *p*-value for the linear regression models after backwards elimination predicting average COF in BCS–CoCr and PBS–CoCr conditions.

Model	Observations (n)	R^2^	Adjusted R^2^	RMSE	F-Statistic	Model *p*-Value
CoCr–BCS COF	11	0.532	0.415	0.047	4.53	0.048
CoCr–PBS COF	22	0.710	0.680	0.060	23.3	<0.001

## Appendix B

**Table A5.** Coefficient of variation (CV, %) of the Wear Score for each HBM.

HBM	CV (%)	Observations (n)
Alumina	0	2
AZ	0.29459	4
CoCr	0.28419	38
Glass	0.15385	4
HXLPE	0.5	3
Hydrogel	0.29017	30
OxZr	0	1
PCU	0.14445	20
PEEK	0.30822	20
PU	0.45356	9
SS	0.24718	18

**Table A6.** Results of linear regression models for the Wear Score versus COF across HBM—lubricant combinations reported with 95% confidence intervals (CI), count (n), and significance. Bold entries indicate statistically significant trends (*p* < 0.05).

HBM	Lubricant	Slope	Lower 95% CI	Upper 95% CI	*p*-Value	Observations (n)
AZ	BCS	−2.63	−11.3	6.05	0.322	4
CoCr	BCS	0.6	−3.48	4.68	0.76	19
CoCr	PBS	−0.509	−3.52	2.5	0.726	19
Glass	DI	−4.22 × 10^−15^	N/A	N/A	N/A	2
HXLPE	BCS	−24.2	−81.2	32.8	0.117	3
Hydrogel	BCS	−0.0138	−4.45	4.42	0.995	14
Hydrogel	DI	0	0	0	N/A	4
Hydrogel	PBS	−1.34 × 10^−14^	−1.34 × 10^−14^	−1.34 × 10^−14^	0	4
Hydrogel	RS	−1.07	−241	239	0.964	3
Hydrogel	SF	18.9	−16.97	54.7	0.152	4
PCU	BCS	0.451	−0.941	1.84	0.468	9
PCU	PBS	0.268	−3.82	4.35	0.881	9
PCU	SF	−4.49 × 10^−15^	N/A	N/A	N/A	2
**PEEK**	**BCS**	**−14.8**	**−25.9**	**−3.83**	**0.014**	**11**
PEEK	PBS	2.04	−0.503	4.58	0.099	9
PU	BCS	23.1	−993	1039	0.821	3
PU	DI	−1.50 × 10^−15^	N/A	N/A	N/A	2
**PU**	**HA**	**5.96**	**3.23**	**8.69**	**0.011**	**4**
SS	BCS	0.41	−2.17	2.99	0.737	15
SS	PBS	−4.04	N/A	N/A	N/A	2

**Table A7.** Model fit statistics including sample size (n), coefficient of determination (R^2^), adjusted R^2^, root mean squared error (RMSE), F-statistic, and overall model *p*-value for the linear regression models predicting wear score in BCS–CoCr and PBS–CoCr conditions.

Model	Observations (n)	R^2^	Adjusted R^2^	RMSE	F-Statistic	Model *p*-Value
CoCr–BCS Wear Score	19	0.294	0.093	0.561	1.46	0.267
CoCr–PBS Wear Score	18	0.308	0.095	0.992	1.45	0.274

**Table A8.** Model fit statistics including sample size (n), coefficient of determination (R^2^), adjusted R^2^, root mean squared error (RMSE), F-statistic, and overall model *p*-value for the linear regression models after backwards elimination predicting wear score in BCS–CoCr and PBS–CoCr conditions.

Model	Observations (n)	R^2^	Adjusted R^2^	RMSE	F-Statistic	Model *p*-Value
CoCr–BCS Wear Score	19	0.179	0.132	0.549	3.71	0.07
CoCr– PBS Wear Score	18	0.205	0.158	0.962	4.37	0.052

## Appendix C

**Table A9.** QUIN [21] risk-of-bias assessment results for the 30 included studies. The table presents each study’s total score and corresponding final percentage score.

Study	Total Score	Final Score (%)
Ajdari [23]	21	87.5
Chan [24]	21	87.5
Covert [47]	16	66.7
Elkington [25]	19	79.2
Elkington [26]	17	70.8
Elkington [27]	19	79.2
Foy [28]	19	79.2
Hu [29]	19	79.2
Kanca [30]	21	87.5
Kanca [31]	21	87.5
Kyomoto [32]	20	83.3
Li [33]	16	66.7
Li [34]	16	66.7
Lizhang [35]	19	79.2
Lizhang [49]	21	87.5
Lu [36]	19	79.2
Luo [37]	21	87.5
McCann [50]	20	83.3
McCann [51]	20	83.3
Morimoto [38]	20	83.3
Northwood [39]	20	83.3
Northwood [22]	20	83.3
Oungoulian [40]	20	83.3
Patel [41]	20	83.3
Qian [42]	20	83.3
Sardinha [43]	20	83.3
Spartacus [48]	21	87.5
Wan [44]	20	83.3
Wan [45]	20	83.3
Zhang [46]	20	83.3

## Abbreviations

AZ: Alumina-Zirconia Composite
COF: Coefficient of Friction
CV: Coefficient of Variation
CoCr: Cobalt-Chromium Alloy
DI: Deionized (water)
HA: Hyaluronic Acid
HBM: Hemiarthroplasty Bearing Material
HXLPE: Highly Cross-Linked Polyethylene
OxZr: Oxidized Zirconium
PCU: Polycarbonate Urethane
PBS: Phosphate-Buffered Saline
PEEK: Polyether Ether Ketone
PU: Polyurethane
RMSE: Root Mean Squared Error
SF: Synovial Fluid
