# Supplementary material for: Friction and Cartilage Wear in Hemiarthroplasty: A Systematic Review of Key Influencing Factors
Source: Lubricants. Author manuscript; Available in PMC 2026 Feb 11. (PMC12889880; doi:10.3390/lubricants14010018)
Supplement: List of Suppl Material [file NIHMS2141622-supplement-List_of_Suppl_Material.docx]

**Supplementary Material 1. Search Strings and Study Characteristics.** This supplementary material summarizes the search strategies and results across multiple databases, as well as key characteristics of the included studies. It provides an overview of the hemiarthroplasty bearing materials tested, study populations, joint types, and tribometer testing configurations.

Supplementary Material Table 1. Pubmed Search Strategy and Results for the Scoping Review on HBMs

Supplementary Material Table 2. Scopus Search Strategy and Results for the Scoping Review on HBMs

Supplementary Material Table 3. Cochrane Search Strategy and Results for the Scoping Review on HBMs

Supplementary Material Table 4. Google Scholar Search Strategy and Results for the Scoping Review on HBM

Supplementary Material Table 5. Hemiarthroplasty Bearing Materials (HBM) Tested Across Studies

Supplementary Material Table 6. Study Source, Ages, and Joint Types Tested Supplementary Material Table 7. Tribometer Configurations and Set Up

Supplementary Material Table 8. Summary of Experimental Testing Parameters For Tribometers Supplementary Material Table 9. Summary of Experimental Testing Parameters For Tribometers

**Supplementary Material 2. Calculations of Testing Input Parameters.** This supplementary material details how total sliding distance, number of reciprocating cycles, and motion frequency were calculated for each study. Calculations were based on reported stroke lengths, sliding velocities, durations, and, where applicable, arc lengths for angular or cross-shear motions. All distances were converted to meters and frequencies to Hz for consistency.

**Supplementary Material 3. Coefficient of Friction Data Set.** Included tables present detailed tribological testing results for various bearing materials used in hemiarthroplasty, including CoCr, PEEK, PU, HXLPE, Hydrogel, Alumina, SS, and Zirconia. Key parameters reported include average coefficient of friction (COF) at multiple time points, contact stress, sliding velocity, lubricant type, and final COF values, providing a comprehensive overview of material performance under experimental conditions.

Supplementary Material Table 10. Tribological Testing Parameters and Coefficient of Friction for all Hemiarthroplasty Bearing Materials

Supplementary Material Table 11. Tribological Testing Parameters and Coefficient of Friction for Hemiarthroplasty Bearing Materials for CoCr

Supplementary Material Table 12. Tribological Testing Parameters and Coefficient of Friction for Hemiarthroplasty Bearing Materials for HXLPE

Supplementary Material Table 13. Tribological Testing Parameters and Coefficient of Friction for Hemiarthroplasty Bearing Materials for Hydrogels

Supplementary Material Table 14. Tribological Testing Parameters and Coefficient of Friction for Hemiarthroplasty Bearing Materials for PCU

Supplementary Material Table 15. Tribological Testing Parameters and Coefficient of Friction for Hemiarthroplasty Bearing Materials for PEEK

Supplementary Material Table 16. Tribological Testing Parameters and Coefficient of Friction for Hemiarthroplasty Bearing Materials for PU

Supplementary Material Table 17. Tribological Testing Parameters and Coefficient of Friction for Hemiarthroplasty Bearing Materials for SS

**Supplementary Material 4. Coefficient of Friction Predictive Modelling.** This supplementary material presents COF predictive modeling results for all available HBM–lubricant combinations. Linear and stepwise regression models were developed where possible, with tables summarizing model coefficients, performance metrics, and statistical tests. Some models were omitted when predictor variables lacked variability, limiting their predictive value. Tables are organized by HBM and lubricant for clarity.

Supplementary Table 18. Hydrogel-BCS Full Model

Supplementary Table 19. Hydrogel-BCS Backward Elimination Model Supplementary Table 20. HXLPE-BCS Full Model

Supplementary Table 21. HXLPE-BCS Backward Elimination Model Supplementary Table 22. PCU-BCS Full Model

Supplementary Table 23. PCU-BCS Backward Elimination Model Supplementary Table 24. PCU-PBS Full Model

Supplementary Table 25. PCU-PBS Backward Elimination Model Supplementary Table 26. PEEK-BCS Full Model

Supplementary Table 27. PEEK-BCS Backward Elimination Model Supplementary Table 28. PEEK-PBS Full Model

Supplementary Table 29. PEEK-PBS Backward Elimination Model

**Supplementary Material 5. Wear Score Data Set.** This dataset provides a summary of tribological testing parameters and the corresponding wear scores across the included studies. Columns include the study reference, average coefficient of friction (COFAVG), lubricant type, material tested, wear score, contact stress (MPa), sliding velocity (cm/s), and sliding distance

(m). Each entry lists the specific experimental conditions alongside the assigned wear score, allowing for comparison of friction and wear behavior across different materials, lubricants, and testing regimes.

Supplementary Material Table 30. Tribological Testing Parameters and Wear Score for all Hemiarthroplasty Bearing Materials

Supplementary Material Table 31. Tribological Testing Parameters and Wear Score for Alumina

Supplementary Material Table 32. Tribological Testing Parameters and Wear Score for Hemiarthroplasty Bearing Materials for AZ

Supplementary Material Table 33. Tribological Testing Parameters and Wear Score for Hemiarthroplasty Bearing Materials for CoCr

Supplementary Material Table 34. Tribological Testing Parameters and Wear Score for Hemiarthroplasty Bearing Materials for Glass

Supplementary Material Table 35. Tribological Testing Parameters and Wear Score for Hemiarthroplasty Bearing Materials for HXLPE

Supplementary Material Table 36. Tribological Testing Parameters and Wear Score for Hemiarthroplasty Bearing Materials for Hydrogels

Supplementary Material Table 37. Tribological Testing Parameters and Wear Score for Hemiarthroplasty Bearing Materials for PCU

Supplementary Material Table 38. Tribological Testing Parameters and Wear Score for Hemiarthroplasty Bearing Materials for PEEK

Supplementary Material Table 39. Tribological Testing Parameters and Wear Score for Hemiarthroplasty Bearing Materials for PU

Supplementary Material Table 40. Tribological Testing Parameters and Wear Score for Hemiarthroplasty Bearing Materials for SS
