# Supplementary material for: Friction and Cartilage Wear in Hemiarthroplasty: A Systematic Review of Key Influencing Factors
Source: Lubricants. Author manuscript; Available in PMC 2026 Feb 11. (PMC12889880; doi:10.3390/lubricants14010018)
Supplement: Suppl Material 1 [file NIHMS2141622-supplement-Suppl_Material_1.docx]

Supplementary Material 1: Search Strings and Study Characteristics

This scoping review was conducted following the Arksey and O'Malley framework. The search strategy was developed in collaboration with research librarians from Rush University and the University of Illinois Chicago (UIC) using a combination of keywords and Medical Subject Headings (MeSH) terms to identify relevant in vitro studies on hemiarthroplasty bearing materials. Searches were performed across PubMed (Table 1), Scopus (Table 2), Google Scholar (Table 3), and the Cochrane Library (Table 4) in December 2024. The strategy comprised four individual search strings, each designed to capture studies related to different aspects of the research question.

Each table presents the individual search strings, the corresponding search results per database, and how these search strings were combined to yield the final set of included studies. The search strings targeted:

1. **Hemiarthroplasty Concepts** – Identifying studies related to hemiarthroplasty procedures.
2. **Cartilage and Cartilage Wear** – Capturing investigations on cartilage degradation or wear in joint simulations.
3. **Hemiarthroplasty Bearing Materials (HBMs)** – Including HBMs commonly used in hemiarthroplasty implants.
4. **In Vitro Models** – Focusing on pre-clinical models assessing cartilage-implant interactions under lubricated tribological conditions.

The final search combined these strings using Boolean operators to refine the results, ensuring comprehensive coverage of studies evaluating the tribological performance of hemiarthroplasty bearing materials

**Supplementary Material Table 1. Pubmed Search Strategy and Results for the Scoping Review on HBMs**

| **#** | **Search String** | **Results** |
| --- | --- | --- |
| 1 | Hemiarthroplast*[tiab] OR "Bearing surface"[tiab] OR "Bearing surfaces"[tiab] OR Biomaterial*[tiab] OR Hemi-Arthroplast*[tiab] OR "Hemi Arthroplasty"[tiab] OR "Hemiarthroplasty"[Mesh] OR "hemiendoprosthes*"[tiab] OR "hemi-endoprosthes*"[tiab] OR "hemi endoprosthes*"[tiab] | 65,632 |
| 2 | cartilage[tiab] OR "Cartilage"[Mesh] | 151,847 |
| 3 | abrasion[tiab] OR damage[tiab] OR damaged[tiab] OR disorder*[tiab] OR erosion[tiab] OR friction[tiab] OR lesion[tiab] OR loss[tiab] OR lost[tiab] OR scratch*[tiab] OR scar*[tiab] OR tear*[tiab] OR torn[tiab] OR wear[tiab] OR wearing[tiab] OR wore[tiab] OR worn[tiab] | 4,260,561 |
| 4 | tissue engineering[tiab] OR "tissue regeneration"[tiab] | 81,164 |
| 5 | (#1 AND #2 AND #3) NOT #4 | 518 |
| 6 | Hemiarthroplast*[tiab] OR "Bearing surface"[tiab] OR "Bearing surfaces"[tiab] OR Biomaterial*[tiab] OR Hemi-Arthroplast*[tiab] OR "Hemi Arthroplasty"[tiab] OR "Hemiarthroplasty"[Mesh] OR "hemiendoprosthes*"[tiab] OR "hemi-endoprosthes*"[tiab] OR "hemi endoprosthes*"[tiab] OR cartilage[tiab] OR "Cartilage"[Mesh] | 214,566 |
| 7 | wear simulator[tiab:~2] OR "joint simulator"[tiab:~2] OR "wear simulation"[tiab:~2] OR "joint simulation"[tiab:~2] OR "wear simulating"[tiab:~2] OR "joint simulating"[tiab:~2] OR "wear simulate"[tiab:~2] OR "joint simulate"[tiab:~2] OR "wear simulated"[tiab:~2] OR "joint simulated"[tiab:~2] | 2,268 |
| 8 | #6 AND #7 | 243 |
| 9 | #5 OR #8 | 753 |
| 10 | #9 AND ("1900/01/01"[Date - Publication] : "2024/12/12"[Date - Publication]) | 735 |

**Table 1.** This table presents the individual search strings used to identify relevant studies, along with the corresponding number of results retrieved from database searches. Boolean operators were used to refine the search, ensuring the inclusion of studies on hemiarthroplasty, cartilage wear, and biomaterials while excluding unrelated topics such as tissue engineering. The final search set was limited to studies published between**1900 and December 12, 2024.**

**Supplementary Material Table 2. Scopus Search Strategy and Results for the Scoping Review on HBMs**

| **#** | **Search String** | **Results** |
| --- | --- | --- |
| 1 | TITLE-ABS-KEY(hemiarthroplast* OR "Bearing surface" OR "Bearing surfaces" OR biomaterial* OR hemi-arthroplast* OR "Hemi Arthroplasty" OR "hemiendoprosthes*" OR "hemi-endoprosthes*" OR "hemi endoprosthes*") | 187,367 |
| 2 | TITLE-ABS-KEY(cartilage) | 181,022 |
| 3 | TITLE-ABS-KEY(abrasion OR damage OR damaged OR disorder* OR erosion OR friction OR lesion OR loss OR lost OR scratch* OR scar* OR tear* OR torn OR wear OR wearing OR wore OR worn) | 11,236,891 |
| 4 | TITLE-ABS-KEY ("tissue engineering" OR "tissue regeneration") | 190,373 |
| 5 | (#1 AND #2 AND #3) NOT #4 | 900 |
| 6 | TITLE-ABS-KEY(Hemiarthroplast* OR "Bearing surface" OR "Bearing surfaces" OR Biomaterial* OR Hemi-Arthroplast* OR "Hemi Arthroplasty" OR "hemiendoprosthes*" OR "hemi-endoprosthes*" OR "hemi endoprosthes*" OR cartilage) | 362,040 |
| 7 | TITLE-ABS-KEY((wear W/2 simulat*) OR (joint W/2 simulat*)) | 12,306 |
| 8 | #6 AND #7 | 508 |
| 9 | #5 OR #8 | 1389 |

**Table 2.** This table outlines the search strategy conducted in **Scopus**, including individual search strings, Boolean operations, and the number of results retrieved. The search focused on **hemiarthroplasty, cartilage wear,** and**biomaterials,** while excluding unrelated fields such as **tissue engineering**. Proximity operators (W/2) were used to refine searches related to **wear and joint simulation**. The final dataset was obtained by combining relevant search results to ensure a comprehensive selection of studies for review.

**Supplementary Material Table 3. Cochrane Search Strategy and Results for the Scoping Review on HBMs**

| **#** | **Search String** | **Results** |
| --- | --- | --- |
| 1 | TITLE-ABS-KEY(hemiarthroplast* OR "Bearing surface" OR "Bearing surfaces" OR biomaterial* OR hemi-arthroplast* OR "Hemi Arthroplasty" OR "hemiendoprosthes*" OR "hemi-endoprosthes*" OR "hemi endoprosthes*") | 187,367 |
| 2 | TITLE-ABS-KEY(cartilage) | 181,022 |
| 3 | TITLE-ABS-KEY(abrasion OR damage OR damaged OR disorder* OR erosion OR friction OR lesion OR loss OR lost OR scratch* OR scar* OR tear* OR torn OR wear OR wearing OR wore OR worn) | 11,236,891 |
| 4 | TITLE-ABS-KEY ("tissue engineering" OR "tissue regeneration") | 190,373 |
| 5 | (#1 AND #2 AND #3) NOT #4 | 900 |
| 6 | TITLE-ABS-KEY(Hemiarthroplast* OR "Bearing surface" OR "Bearing surfaces" OR Biomaterial* OR Hemi-Arthroplast* OR "Hemi Arthroplasty" OR "hemiendoprosthes*" OR "hemi-endoprosthes*" OR "hemi endoprosthes*" OR cartilage) | 362,040 |
| 7 | TITLE-ABS-KEY((wear W/2 simulat*) OR (joint W/2 simulat*)) | 12,306 |
| 8 | #6 AND #7 | 508 |
| 9 | #5 OR #8 | 1389 |
| 10 | filtered to Publication date ending in 2024 | 1389 |

**Table 3.** This table details the Cochrane Library search strategy, including individual search strings, Boolean operations, and retrieved results. The search targeted studies related to hemiarthroplasty, cartilage wear, and biomaterials, while excluding unrelated fields such as tissue engineering. Proximity operators (W/2) were used to refine searches on wear and joint simulation. The final dataset was obtained by applying a publication date filter through 2024 to ensure relevant study selection.

**Supplementary Material Table 4. Google Scholar Search Strategy and Results for the Scoping Review on HBM**

| **#** | **Search String** | **Results** |
| --- | --- | --- |
| 1 | (Hemiarthroplasty OR "Bearing surface" OR Biomaterial*) (cartilage) (abrasion OR damage OR disordered OR erosion OR friction OR lesion OR loss OR lost OR scratch OR scar OR tear OR torn OR wear OR wore OR worn) (wear simulator OR joint simulator) | 50 |

Table 4. This table presents the search strategy used in Google Scholar, including the combination of keywords and Boolean operators to identify relevant studies. The search focused on hemiarthroplasty, cartilage wear, and biomaterials while incorporating terms related to wear simulation. The top 50 results were selected based on the search query.

**Supplementary Material Table 5. Hemiarthroplasty Bearing Materials (HBM) Tested Across Studies**

| **Study ID** | **HBM Tested** |
| --- | --- |
| Ajdari 2020 | CoCr alloy, AL2O3, PCU |
| Chan 2011 | Al2O3, CoCr, UHMWPE, SS |
| Covert 2001 | GT hydrogel |
| Cowie 2021 | Stainless steel |
| Elkington 2023 | Untreated PEEK, SPMK-g-PEEK, CoCrMo alloy |
| Elkington 2024a | SPMK-g-PEEK, PEEK |
| Elkington 2024b | PEEK, SPMK-g-PEEK |
| Foy 1999 | Borosilicate glass, Borosilicate glass coated with DPPC, PU elastomer, Surface-coated PU |
| Kanca 2018a | PVA/PVP blend hydrogels (lower) |
| Kanca 2018b | PCU, SS |
| Kyomoto 2010 | Co–Cr–Mo with MPC polymer (grafting, dip coating), CoCrMo variations |
| Li 2010 | PVA hydrogel, SS |
| Li 2016 | PVA hydrogel |
| Lizhang 2011 | CoCr alloy |
| Lizhang 2013 | Custom-made CoCr femoral heads |
| Lu 2022 | CoCrMo, PEEK, HXLPE |
| Luo 2010 | PU (different moduli), SS |
| McCann 2008 | Stainless steel |
| McCann 2009 | Stainless steel |
| Morimoto 2014 | PVA-H/Water, PVA-H/DMSO |
| Northwood 2007a | Biphasic hydrogel, SS |
| Northwood 2007b | Biphasic hydrogel, SS, Silicone, PU |
| Oungoulian 2015 | Low carbon CoCr, High carbon CoCrHC, SS |
| Patel 1997 | CoCr, OxZr |
| Qian 2019 | Glass |
| Sardinha 2013 | PVA |
| Spartacus 2017 | Biozyrs/aluminia/zirconium |
| Wan 2020 | PCU nanostructured adhesive coatings (various formulations) |
| Wan 2021 | PCU, PCU nanostructured adhesive coatings (various formulations) |
| Zhang 2021 | PEEK, CoCrMo alloy, Zirconia ceramic |

Table 5. This table lists the bearing materials tested in various studies on hemiarthroplasty, along with their corresponding Study ID. The HBMs include a variety of metals, ceramics, polymers, hydrogels, and composites used in the evaluation of their interactions with cartilage in the context of hemiarthroplasty.

**Supplementary Material Table 6. Study Source, Ages, and Joint Types Tested**

| **Study ID** | **Source** | **Age** | **Joint Type** |
| --- | --- | --- | --- |
| Ajdari 2020 | human | not specified | femoral head |
| Bowland 2018 | porcine | 4-6 mo. | tibiofemoral joints |
| Chan 2011 | bovine | 1–3 wks | distal femoral medial condyle |
| Covert 2001 | bovine | not specified | articular cartilage not specified |
| Elkington 2023 | bovine | 24-48 mo | patellofemoral grooves |
| Elkington 2024a | bovine | 12-24 mo | patellofemoral grooves |
| Elkington 2024b | bovine | 24 mo | patellofemoral grooves |
| Foy 1999 | bovine | adult | metacarpal bones |
| Kanca 2018a | ovine | 18-24 mo | medial and lateral femoral condyles |
| Kanca 2018b | ovine | 18-24 mo | patellofemoral joint/condyle |
| Kyomoto 2010 | porcine | 6-9 mo | flat part of the ankle joint |
| Li 2010 | human | not specified | femoral condyle |
| Li 2016 | bovine | 18 mo | femoral condyle |
| Lizhang 2011 | bovine | 18 mo | patello-femoral groove |
| Lizhang 2013 | porcine | 6 mo | Acetabulum |
| Lu 2022 | bovine | 18 mo. | patellofemoral groove |
| Luo 2010 | bovine | 18- 24 mo. | medial femoral condyle |
| McCann 2008 | bovine | 18-24 mo | Medial femoral condyles |
| McCann 2009 | bovine | 18-24 mo | Medial femoral condyles |
| Morimoto 2014 | porcine | not specified | Knee (not specified) |
| Northwood 2005 | bovine | not specified | patella-femoral canal |
| Northwood 2007a | bovine | 18 mo. | patellofemoral canal |
| Northwood 2007b | bovine | 18 mo. | femur (hip joint) |
| Oungoulian 2015 | bovine | 2–3 mo. | tibiofemoral joint ** |
| Patel 1997 | bovine | not specified | femoral condyles |
| Qian 2019 | bovine | 18-24 mo | femora (not specified) |
| Sardinha 2013 | bovine | not specified (adult) | Knee (not otherwise specified) |
| Spartacus 2017 | porcine | not specified | phalangeal joint |
| Wan 2020 | bovine | not specified | not specified |
| Wan 2021 | bovine | 24 mo | vin |
| Zhang 2021 | bovine | 18 mo. | femur (hip joint) |

**Oungoulian 2015 - tibial plateaus and opposing femoral condyle - tibial plateau)

Table 6. This table provides a summary of specimen types, ages, and joint types across various studies focused on hemiarthroplasty. The studies utilize different species and ages, ranging from bovine and porcine models to human specimens, covering multiple joint types for testing HBMs.

**Supplementary Material Table 7. Tribometer Configurations and Set Up**

| **Study ID** | **Testing Configuration** | **Testing Apparatus** | **Motion** | **Cartilage shape** |
| --- | --- | --- | --- | --- |
| Ajdari 2020 | Pin-on-disc | Custom multiaxial testing rig | Reciprocal sliding | Pin |
| Chan 2011 | Pin-on-disc | Not specified | Reciprocal sliding motion | Pin |
| Covert 2001 | Pin-on-plate | Not specified | Reciprocal sliding | pin |
| Elkington 2023 | Pin-on-plate | Bruker UMT Tribolab | Reciprocal sliding | Pin |
| Elkington 2024 | Pin-on-plate | Bruker UMT Tribolab | Reciprocal sliding | pin |
| Elkington 2024 | Pin--on-disc | Micro Traction Machine, PCS Instruments, UK | Reciprocal sliding | pin |
| Foy 1999 | Pin-on-plate | Custom jig | Reciprocal sliding | pin |
| Hu 2023 | Ball-on-pin | UMT, Bruker, USA | Reciprocal sliding | pin |
| Kanca 2018a | Pin-on-disc | Custom jig | Multidirectional reciprocating | Pin |
| Kanca 2018b | Pin-on-plate | Custom jig | Multidirectional reciprocating | pin |
| Kanca 2018b | Pin-on-plate | Custom jig | Multidirectional reciprocating | plate |
| Kyomoto 2010 | Pin-on-plate | Tribostation 32; Shinto Scientific Co., | Reciprocal sliding | pin |
| Li 2010 | Pin-on-plate | UMT-2 Micro-Tribometer (CETR INC.) | Reciprocal sliding | Pin |
| Li 2016 | Pin-on-plate | home-built tribometer | Reciprocal sliding | pin |
| Lizhang 2011 | Pin-on-plate | Custom jig | Reciprocal sliding | pin |
| Lu 2022 | Pin-on-disc | Rtec Multi Function Tribometer - 5000B | Multidirectional reciprocating | Pin |
| Lizhang 2013 | Ball-on-joint | Pendulum friction simulator (Simulator solutions, Manchester UK) | acetabular joint | n/a |
| Luo 2010 | Plate-on-joint | Commercial Prosim pendulum friction simulator (Simulation Solutions, UK) | knee joint femoral condyle | n/a |
| McCann 2008 | Plate-on-joint | Commercial Prosim pendulum friction simulator (Simulation Solutions, UK) | knee joint femoral condyle | n/a |
| Morimoto 2014 | Pin--on-disc | self- made tribometer | Continuous rotational | pin |
| Northwood 2005 | Pin-on-plate | Simple geometry simulator (no other details provided) | Reciprocal sliding | Pin |
| Northwood 2007 | Pin-on-plate | Custom (schematic) | Reciprocal sliding | Pin |
| Northwood 2007 | Pin-on-plate | Custom (schematic) | Reciprocal sliding | Pin |
| Oungoulian 2015 | Pin-on-disc | Custom two-axis loading device equipped with a six-degrees-of-freedom load cell (JR3 Inc. #20E12A4, Woodland, CA) | Reciprocal sliding | Disc |
| Patel 1997 | Pin-on-disc | Custom jig modified from Implant Sciences Company, Danvers, MA, USA (no schematic or photo) | Continuous rotational sliding motion | Pin |
| Qian 2019 | Pin-on-plate | UMT-2 test machine (Center for Tribology, USA) | Reciprocal sliding | pin |
| Sardinha 2013 | Pin-on-disc | Plint TE67/R tribometer (Phoenix Tribology Ltd, UK). | Reciprocal sliding | disc |
| Spartacus 2017 | Pin-on-plate | Custom | Reciprocal sliding | pin |
| Wan 2020 | Pin-on-disc | universal mechanical tester (UMT-3, CETR Inc., USA | Reciprocal sliding | pin |
| Wan 2021 | Pin-on-disc | universal mechanical tester (UMT-3, CETR Inc., USA) | Reciprocal sliding | pin |
| Zhang 2021 | Pin-on-disc | Commercial Rtec Instrument Technology Co., Ltd. (Nanjing)). | Continuous sliding | Disc |

Table 7. This table outlines the testing configurations, apparatus, motion types, and cartilage shapes used in various studies examining the tribometer behavior of HBM implants in hemiarthroplasty research. The table provides a detailed overview of the experimental setups and the specific configurations employed for each study to simulate the mechanical interaction between HBMs and cartilage under different loading and motion conditions.

**Supplementary Material Table 8. Summary of Experimental Testing Parameters For Tribometers**

| **Study ID** | **Technical Contact Stress (MPa)** | **Velocity (mm/s)** | **Frequency (Hz)** | **Testing time (s)** | **Distance (m)** | **Cycles** |
| --- | --- | --- | --- | --- | --- | --- |
| Ajdari 2020 | 0.3 | 4 | 1 | 3600* | 14.45 * | 3600 |
| Chan 2011 | 0.1 | 0.5 | 0.032* | 3600 | 1.8* | 115* |
| Covert 2001 | 0.5 | 50 | 1* | 2700 | 135* | 2700 |
| Elkington 2023 | 0.75 | 10 | 0.25, 0.5* | 9000 | 90 | 2250, 4500 |
| Elkington 2024a | 0.75 | 10 | 0.5* | 1800 | 18 | 900* |
| Elkington 2024b | 0.25-2.21 | 1-10 | 0.005 -0.5* | 1800 | 1.8 -18* | 9-900* |
| Foy 1999 | 1.197 | 25-50 | 1.25-2.5* | 300 | 60* | 500 |
| Hu 2023 | 0.8 | 10 | 0.5 | 11400 | 114* | 5,700* |
| Impergre 2023 | 1.6 | 16.76* | 0.5 | 54000 | 903.84* | 27000* |
| Kanca 2018a | 0.58- 1.41 | 25 | 1 | 54,000 | *1703 | 54,000 |
| Kanca 2018b | 0.81-1.41 | 25 | 1 | 3600, 54000 | *113.56, 1703 | 3600, 54000 |
| Kyomoto 2010 | 0.49 to 9.80 | 50 | 1 | 5000 | 250* | 5000 |
| Li 2010 | 0.4 | 2 | 0.5* | 3600 | 7.2 m* | 1800* |
| Li 2016 | 0.4 | 10 | 1.11, 2.22* | 7200 | 9.18, 18.36* | 7992, 15984 |
| Ling 2015 | 0.027* | 0.599* | 0.0159* | 90 | 0.0539* | 1.43* |
| Lizhang 2011 | 0.5 - 16 | 4, 8 | 1 | 3600, 86400 | *14.4- 691.2 | *3600, 86400 |
| Lizhang 2013 | 5.6, 6.7, 7.4, 10.6 | 8.38, 8.9, 9.15, 9.41 | 1 | 7200 | 60.34, 64.08, 65.88, 67.27 | 7200 |
| Lu 2022 | 1-3 | 10 | *0.25 | 5400 | 80.96, 161.91* | 1,350, 2700* |
| Luo 2010 | 4.039- 23.297 | 30.63-33.85* | 1 | 3600 | 110.26-121.87* | 3600 |
| McCann 2008 | 8.9, 31 | 17.82 | 1 | 300, 3600 | 5.4, 64.2 | 300, 3600 |
| McCann 2009 | 3, 4, 8, 14.9, 23.5 | 17.82 | 1 | 3600 | 64.2 | 3600 |
| Northwood 2005 | 0.5 | 4* | 0.4 | 21,600 | 86.4* | 8,640* |
| Northwood 2007a | 0.5 | 4 | 0.4 | 28,800 | 616* | 28,800* |
| Northwood 2007b | 0.5 | 4 | 0.2 | 7200, 14400 | 57.6, 576* | 2880, 28800 |
| Oungoulian 2015 | 0.18 | 1 | 0.1 | 14,400 | 14.4* | 1,440* |
| Patel 1997 | 0.49-0.78 | 100 | 2.27* | 90,000* | 9000 | 204624* |
| Sardinha 2013 | 1-5 | 16 | 1 | 7200 | 115.2* | 7200* |
| Schwartz 2007 | 0.533, 0.447 | 21 | 0.82 | 121,951.22 | 2,560 | 100000 |
| Spartacus 2017 | 1,2, 6.67 | 1 | 0.2, 1 | 2000, 100000 | 2.4,4, 12 | 400, 2000 |
| Wan 2020 | 0.4 | 4 | 0.2 | 3600 | 14.4* | 720 |
| Wan 2021 | 0.4, 4 | 4 | 0.2 | 3600 | 14.4* | 720 |
| Zhang 2021 | 1-3 | 2 | unidirectional | 1800-43200 | 3.6- 86.4 | unidirectional |

Table 8. This table provides a comprehensive summary of the testing parameters used across various studies, including technical contact stress, velocity, frequency, testing time, distance, and cycles. Asterisks (*) indicate values that were calculated, with detailed calculations available in the supplementary material.

**Supplementary Material Table 9. Summary of Experimental Testing Parameters For Tribometers**

| **Study ID** | **Lubricant** |
| --- | --- |
| Ajdari 2020 | BCS |
| Chan 2011 | PBS |
| Covert 2001 | 50% DI, BCS |
| Elkington 2023 | PBS |
| Elkington 2024 (a) | PBS or NaCl |
| Elkington 2024 (b) | PBS |
| Foy 1999 | Deionized water |
| Kanca 2018 | 30% (v/v) bovine calf serum (BCS) |
| Kanca 2018 | BCS was diluted with deionised water (3:7 by volume) (SF/ 30% BCS) |
| Kyomoto 2010 | * |
| Li 2010 | RS |
| Lizhang 2011 | PBS |
| Lizhang 2013 | PBS |
| Lu 2022 | BCS with additives (including EDTA) |
| Luo 2010 | BCS (25%) w/ PBS |
| McCann 2008 | 25% Bovine calf serum in saline (0.1% sodium azide) |
| McCann 2009 | 25% Bovine calf serum in saline (0.1% sodium azide) |
| Northwood 2005 | RS |
| Northwood 2007 | 25% BCS w/ 0.1% sodium azide |
| Northwood 2007 | RS or 25 per cent bovine serum containing protein at 16–18 mg/l with 0.1 per cent sodium azide |
| Oungoulian 2015 | PBS |
| Patel 1997 | BCS |
| Qian 2019 | 50% bovine calf serum |
| Sardinha 2013 | DI water and PBS |
| Spartacus 2017 | 100% concentrated of fetal bovine serum |
| Wan 2020 | Synovial Fluid |
| Wan 2021 | PBS |

Table. 9 Lubricants utilized in in vitro cartilage studies. The table lists the study IDs along with the respective lubricants employed during testing. Some studies used standard solutions such as PBS, BCS, or Ringer's solution, while others incorporated more specific media like hyaluronic acid (HA), culture media, or a combination of solutions with additives. The specific lubricant used may influence the tribological properties and outcomes in cartilage testing, with some studies providing detailed formulations or concentrations.

*Pure water, mixture of 25 vol% bovine serum (BS), 20mM/L of ethylene diamine tetraacetic acid (EDTA), 0.1 mass% sodium azide, and the BS mixture containing 0.02 mass% MPC polymer (PMB30 (Mw 1⁄4 5.0 104), PMSi90 (Mw 1⁄4 9.8 104), and PMPC (Mw 1⁄4 1.0 105))

**ASTM F732-­ 17 under a sterile hood to reduce the risk of airborne contamination. Newborn calf serum (16010159; Thermo Fisher Scientific, USA) was diluted with autoclaved deionized water to achieve a protein concentration of 20 g/l,32 mimicking the protein concentration in human synovial fluid. Next, 15 ppm ProClin 300 (48,912 U; Sigma-­ Aldrich, USA) and 7 g/l ethylenediaminetetraacetic acid (EDTA) (B8R04080; Philip Harris)
