# Supplementary material for: Friction and Cartilage Wear in Hemiarthroplasty: A Systematic Review of Key Influencing Factors
Source: Lubricants. Author manuscript; Available in PMC 2026 Feb 11. (PMC12889880; doi:10.3390/lubricants14010018)
Supplement: Suppl Material 2 [file NIHMS2141622-supplement-Suppl_Material_2.docx]

**Description of Calculations**

For each study, we extracted the relevant parameters for reciprocating or sliding motion, including stroke length, sliding speed or velocity, duration of testing, and number of cycles where provided. Using these parameters, we calculated the following:

1. **Total Number of Cycles:**
   When not explicitly provided, the total number of cycles was calculated by dividing the total distance traveled by the total distance per cycle. For reciprocating motion, the distance per cycle was considered as the forward plus backward stroke (i.e., twice the stroke length).
2. **Total Distance Traveled:**
   The total sliding distance was calculated as the product of the total number of cycles and the distance per cycle. In cases where only sliding speed and test duration were given, total distance was computed as the product of sliding velocity and total test time.
3. **Frequency of Reciprocation:**
   Frequency was calculated as the number of cycles divided by the total test duration (in seconds), yielding cycles per second (Hz). When velocity and stroke length were known but frequency was not reported, the time per cycle was first calculated as the total distance per cycle divided by the sliding speed, and frequency was then determined as the reciprocal of the cycle time.
4. **Additional Considerations:**
   - For tests involving cross-shear or angular motion, arc length was calculated from the radius and angular displacement using standard geometric relations (arc length = radius × angle in radians) and incorporated into the total distance per cycle.
   - Distances were converted to meters for consistency across studies.
   - For tests reporting variable velocities or multiple segments, calculations were performed separately for each segment, and the total distance or average frequency was determined accordingly.

This approach allowed consistent extraction and standardization of motion parameters across multiple studies for comparative purposes.

**Adjari2020 [26]**

Given:

- Sliding speed: 4 mm/s
- Stroke length: 2 mm (total distance per cycle is 4 mm, since one cycle includes forward and backward motion)
- Frequency: 1 Hz (1 complete cycle per second)
- Duration: 3,613.476720829656 seconds (approximately 1 hour from Matlab)

1. Number of Cycles:

Since the frequency is 1 Hz, meaning 1 cycle per second, the number of cycles can be directly calculated as:

Number of cycles=Frequency×Time

cycles=1×3,613.476720829656≈3,613.48 cycles

So, approximately 3,613.48 cycles were completed during the test.

2. Total Distance Traveled:

Each cycle includes forward and backward motion, so the total travel distance per cycle is 4 mm. The total distance traveled can be calculated as:

Total distance=Number of cycles×Total travel distance per cycle

Total distance=3,613.48×4mm=14,453.91mm

Converting to meters:

14,453.91 mm=14.45 meters

Summary:

- Total distance traveled: 14.45 meters
- Number of cycles: 3,613.48 cycles

**Chan 2011 [27]**

1. Total Distance Traveled:

Distance=Sliding speed×TimeDistance=Sliding speed×TimeDistance=0.5 mm/s×3600 s=1800 mm=1.8 m

So, the total distance traveled is 1.8 meters

2. Number of Reciprocating Cycles:

The number of cycles should be calculated by dividing the total distance traveled by twice the stroke length (since each cycle includes a forward and backward stroke).

Number of cycles=Total distance traveled/2×Stroke length

Number of cycles=1800 mm/2×7.85 mm=180015.7≈114.65 cycles

So, the number of reciprocating cycles is about 114.65 cycles

3. Frequency:

The frequency is the number of cycles per second, which is calculated as:

Frequency=Number of cycles/Total time

​Frequency=114.65 cycles3600 seconds≈0.03185 Hz

So, the frequency is about 0.032 Hz

Summary of Results:

- Total distance traveled: 1.8 meters.
- Number of reciprocating cycles: ~115 cycles.
- Frequency: ~0.032 Hz (cycles per second).

**Covert 2001 [50]**

1. Given Data:

- Sliding speed = 0.05 m/s
- Total cycles = 2,700
- Time = 45 minutes = 2,700 seconds
- Reciprocating motion (back-and-forth per cycle)

2. Reciprocating Motion Distance:

The sliding speed of 0.05 m/s represents the peak speed, which is typically the speed of movement in one direction. Since the motion is reciprocating, we’ll assume this is the speed of a single stroke.

Thus, the total distance traveled per cycle will be twice the distance of a single stroke (back-and-forth).

2.1. Calculate Stroke Length:

If the sliding speed is 0.05 m/s (which is per second), and the frequency is 1 Hz (one full cycle per second), then the stroke length LL (distance traveled in one direction) can be calculated as:

Stroke Length=Sliding Speed2=0.05 m/s2=0.025 m=25 mm

This means that in each cycle, the pin travels 25 mm forward and 25 mm back, making the total distance per cycle 50 mm.

2.2. Calculate Total Distance:

Total distance traveled for all cycles in the test can be calculated as:

Total Distance=Distance per cycle×Total cycles

Total Distance=50mm×2700cycles=135000mm=135m

Summary (Reciprocating Motion):

- Stroke Length: 25 mm (forward or backward)
- Total Distance Traveled: 135 meters (back-and-forth combined)
- Frequency: 1 Hz
- Velocity: 50 mm/s

**Elkington 2023 [28]**

1. Given Data:

SPMK-g-PEEK and PEEK Plates:

- Stroke length: 20 mm (0.02 m)
- Number of cycles: 2,250 cycles

CoCr Plates:

- Stroke length: 10 mm (0.01 m)
- Number of cycles: 4,500 cycles

Common for Both Samples:

- Velocity: 10 mm/s (0.01 m/s)
- Total sliding distance: 90 m
- Displacement profile: Sawtooth

2. Calculations:

We will calculate the frequency for both sample tests based on the given sliding distance, velocity, and number of cycles.

2.1. Time Calculation:

The time for each test can be calculated using the total sliding distance and velocity. Since the total sliding distance is given as 90 m and the velocity is 10 mm/s (or 0.01 m/s), we find the total time required for each test:

Time=Total sliding distanceVelocity=90 m0.01 m/s=9000 seconds

Thus, the time for each test is 9,000 seconds.

2.2. Frequency Calculation for SPMK-g-PEEK and PEEK Plates:

- Stroke length = 0.02 m
- Number of cycles = 2,250 cycles
- Total time = 9,000 seconds

Frequency=Total cyclesTotal time=2250 cycles9000 seconds=0.25 Hz

2.3. Frequency Calculation for CoCr Plates:

- Stroke length = 0.01 m
- Number of cycles = 4,500 cycles
- Total time = 9,000 seconds

Frequency=Total cyclesTotal time=4500 cycles9000 seconds=0.5 Hz

Summary:

- SPMK-g-PEEK and PEEK Plates: 0.25 Hz
- CoCr Plates: 0.5 Hz

**Elkington 2024 [29]**

This study examined a variety of permutations to be able to compare most consistently across groups, the testing set up that focused on reciprocating linear motion was selected for extracting output parameters.

Given:

- Reciprocating Velocity: 10 mm/s
- Sliding Distance: 20 mm (for each cycle)
- Duration of Sliding Phase: 1800 s
- Total Sliding Distance Achieved: 18 m (or 18,000 mm)

Calculations:

1. Number of Cycles:
   Number of Cycles=Total SlidingDistance/Sliding Distance per Cycle

Number of Cycles=18,000 mm/20 mm=900 cycles

1. Frequency: Frequency is defined as the number of cycles per second.

Frequency (Hz)=Number of Cycles/Total Time (s)

Frequency=900 cycles/1800 s=0.5 Hz

Summary:

- Number of Cycles: 900 cycles
- Frequency of Reciprocating Motion: 0.5 Hz

Given Parameters:

- Sliding Speeds (ν): 0.1, 0.5, 1, 2, 5, and 10 mm/s
- Sliding Distance (one-way): 20 mm
- Total Duration: 1800 seconds

Calculations:

1. Distance Traveled per Speed: The distance traveled can be calculated for each speed based on the formula:
   Distance Traveled=Speed×TimeDistance Traveled=Speed×Time
   Since each cycle covers a sliding distance of 20 mm (one way), we will calculate the distance traveled for each speed:
2. Number of Cycles: The number of cycles can be calculated as:
   Number of Cycles=Distance TraveledSliding Distance per Cycle=Distance Traveled20 mmNumber of Cycles=Sliding Distance per CycleDistance Traveled​=20 mmDistance Traveled​

Calculating for Each Speed:

| Speed (mm/s) | Distance Traveled (mm) | Number of Cycles |
| --- | --- | --- |
| 0.1 | 0.1×1800=180 mm | 180/20=9 |
| 0.5 | 0.5×1800=900 mm | 900/20=45 |
| 1.0 | 1.0×1800=1800 mm | 1800/20=90 |
| 2.0 | 2.0×1800=3600 mm | 3600/20=180 |
| 5.0 | 5.0×1800=9000 mm | 9000/20=450 |
| 10.0 | 10.0×1800=18000 mm10.0×1800=18000 mm | 18000/20=900 |

Summary:

- At Speed 0.1 mm/s:
  - Distance Traveled: 180 mm
  - Number of Cycles: 9
- At Speed 0.5 mm/s:
  - Distance Traveled: 900 mm
  - Number of Cycles: 45
- At Speed 1.0 mm/s:
  - Distance Traveled: 1800 mm
  - Number of Cycles: 90
- At Speed 2.0 mm/s:
  - Distance Traveled: 3600 mm
  - Number of Cycles: 180
- At Speed 5.0 mm/s:
  - Distance Traveled: 9000 mm
  - Number of Cycles: 450
- At Speed 10.0 mm/s:
  - Distance Traveled: 18000 mm
  - Number of Cycles: 900

Calculating Frequency for Each Speed:

| Speed (mm/s) | Number of Cycles | Frequency (Hz) |
| --- | --- | --- |
| 0.1 | 9 | 9/1800=0.005 Hz |
| 0.5 | 45 | 45/1800=0.025 Hz |
| 1.0 | 90 | 90/1800=0.05 Hz |
| 2.0 | 180 | 180/1800=0.1 Hz |
| 5.0 | 450 | 450/1800=0.25 Hz |
| 10.0 | 900 | 900/1800=0.5 Hz |

Summary of Frequencies:

- At Speed 0.1 mm/s: Frequency = 0.005 Hz
- At Speed 0.5 mm/s: Frequency = 0.025 Hz
- At Speed 1.0 mm/s: Frequency = 0.05 Hz
- At Speed 2.0 mm/s: Frequency = 0.1 Hz
- At Speed 5.0 mm/s: Frequency = 0.25 Hz
- At Speed 10.0 mm/s: Frequency = 0.5 Hz

**Foy 1999 [31]**

Given Parameters:

- Total Sliding Distance: 60 m (or 60,000 mm)
- Entraining Velocities:
  - Initial velocity: 25 mm/s for the first 250 cycles
  - Subsequent velocity: 50 mm/s for the next 250 cycles
- Total Cycles: 250 + 250 = 500 cycles

Calculations:

1. Total Time for Each Segment:
   - For the first 250 cycles at 25 mm/s:
     Distance for 250 cycles=250×20 mm=5000 mm
   - Time=DistanceVelocity=5000 mm/25 mm/s=200 s
   - For the next 250 cycles at 50 mm/s:
   - Distance for 250 cycles=250×20 mm=5000 mm
   - Time=5000 mm/50 mm/s=100 s
2. Total Time:
   Total Time=200 s+100 s=300 sTotal Time=200 s+100 s=300 s
3. Frequency Calculation:
   Frequency (Hz)=Total Cycles/Total Time (s)=500 cycles/300 s≈1.67 Hz

Summary of Frequency:

- The frequency for the entire sliding distance of 60 m, with the initial entraining velocity of 25 mm/s for the first 250 cycles and 50 mm/s for the next 250 cycles, is approximately 1.67 Hz.

The frequency remains constant throughout the 500 cycles, as the total number of cycles and total time are used to calculate it.

Range of Frequency:

- Minimum Frequency: At 25 mm/s, the time for the initial segment gives an effective frequency of ≈1.25 Hz≈1.25Hz for that segment.
- Maximum Frequency: At 50 mm/s, the effective frequency is ≈2.5 Hz≈2.5Hz for that segment.

Thus, the overall range of frequency during the entire process is approximately 1.25 Hz to 2.5 Hz.

Given Data:

1. Initial Segment:
   - Velocity: 25 mm/s
   - Cycles: 250 cycles
   - Distance per Cycle: 20 mm
2. Calculation for Time:
   Total Distance for 250 cycles=250×20 mm=5000 mm
3. Time for Initial Segment=5000 mm25 mm/s=200 s

Subsequent Segment:

- - Velocity: 50 mm/s
  - Cycles: 250 cycles
  - Distance per Cycle: 20 mm

1. Calculation for Time:
   Total Distance for 250 cycles=250×20 mm=5000 mm
2. Time for Subsequent Segment=5000 mm50 mm/s=100 s

Total Duration of the Test:

Total Duration=Time for Initial Segment+Time for Subsequent Segment=200 s+100 s=300 s

Conclusion:

The total duration of the test is 300 seconds.

**Hu 2023 [32]**

Given:

- Velocity of sliding: 10 mm/s
- Distance per stroke: 10 mm (each direction in the reciprocating motion)
- Total test time: 3 hours + 10 minutes = 190 minutes = 11,400 seconds

1. Frequency:

- Each full cycle (back and forth) is 20 mm (since one stroke is 10 mm, round trip is 20 mm).
- The velocity is 10 mm/s, so the time per stroke is:Time per stroke=10 mm/10 mm/s=1 second
- Thus, the time for a full cycle (back and forth) is:Time per cycle=2 seconds
- The frequency is the reciprocal of the time per cycle:Frequency=12 seconds=0.5 Hz (This means 0.5 cycles per second.)

2. Number of cycles:

- Total time for the test is 11,400 seconds.
- The number of cycles is:Number of cycles=11,400 seconds2 seconds per cycle=5,700 cycles

3. Total distance traveled:

- Each full cycle covers 20 mm, so the total distance traveled is:Total distance=5,700 cycles×20 mm=114,000 mm=114 meters

Summary:

- Frequency: 0.5 Hz (cycles per second)
- Number of cycles: 5,700 cycles
- Total distance traveled: 114 meters

**Kanca 2018 [33]**

Given Data:

- Stroke length: 25 mm
- Mean sliding speed: 25 mm/sec
- Reciprocating frequency: 1 Hz (1 cycle per second)
- Cross shear angle: ±15°
- Radius of circular path: 12.5 mm (half of the stroke length)
- Duration: 15 hours = 54,000 seconds
- Total cycles: 54,000 cycles

Step 1: Calculate the Effective Sliding Path with Cross Shear

In reciprocating motion with a cross shear angle, the sliding path isn't a perfect straight line, but rather an arc. The cross shear introduces some angular deviation, creating a curved path instead of a purely linear motion.

To calculate the distance traveled along the arc, use the arc length formula:

Arc Length=θ×rArc Length=θ×r

Where:

- θθ is the angular displacement in radians,
- rr is the radius of the sliding motion (in this case, 12.5 mm).

Convert the cross shear angle to radians:

θ=15∘×π180=0.2618 radiansθ=15∘×180π​=0.2618radians

Now, the arc length per stroke (forward or backward) is:

Arc Length per stroke=θ×r=0.2618×12.5 mm=3.2725 mm

But this is only part of the total distance. Since each cycle includes a forward and backward motion, the total arc length per cycle is:

Arc Length per cycle=2×3.2725 mm=6.545 mm

However, we still need to add the linear distance traveled by the reciprocating motion. The linear distance per cycle is simply the forward and backward stroke (25 mm total):

Linear Distance per cycle=2×12.5 mm=25 mm

Step 2: Total Distance Per Cycle

To get the total distance per cycle, we add the arc distance due to cross shear and the linear distance:

Total Distance per cycle=25 mm+6.545 mm=31.545 mm

Step 3: Total Distance Traveled

Now, calculate the total distance traveled over 54,000 cycles:

Total Distance Traveled=31.545 mm/cycle×54,000 cycles=1,703,430 mm

Convert this to meters:

Total Distance Traveled=1,703,430 mm=1,703.43 meters

**Kanca 2018 [34]**

The test configuration 1 was 15 hs at the same as Kanca 2018 [33] and the second test configuration was for 1 h below.

Given Data for 1 Hour:

- Stroke length: 25 mm (total sliding distance in one cycle)
- Mean sliding speed: 25 mm/sec
- Reciprocating frequency: 1 Hz (1 cycle per second)
- Cross shear angle: ±15°
- Duration: 1 hour = 3600 seconds
- Total cycles: 3600 cycles (since the frequency is 1 Hz)

Step 1: Convert the Cross Shear Angle to Radians

The angular displacement in radians:

θ=15∘×π180=0.2618 radiansθ=15∘×180π​=0.2618radians

Step 2: Calculate Arc Length per Stroke

Using the radius of the stroke (12.5 mm, which is half of the stroke length):

Arc Length per stroke=θ×r=0.2618×12.5 mm=3.2725 mm

The total arc length per cycle (forward and backward) is:

Arc Length per cycle=2×3.2725 mm=6.545 mm

Step 3: Calculate the Total Linear Distance per Cycle

The linear distance per cycle (forward and backward motion) is:

Linear Distance per cycle=25 mm

Step 4: Calculate Total Distance per Cycle

The total distance per cycle, combining both the arc distance and the linear distance, is:

Total Distance per cycle=25 mm+6.545 mm=31.545 mm

Step 5: Calculate Total Distance Traveled in 1 Hour

Now, calculate the total distance traveled over 3600 cycles:

Total Distance Traveled=31.545 mm/cycle×3600 cycles=113,558 mm

Convert this to meters:

Total Distance Traveled=113,558 mm/1000=113.56 meters

Final Answer:

The total distance traveled during 1 hour of reciprocating motion is approximately 113.56 meters.

**Li 2010 [36]**

Given Data:

- Sliding speed: 2 mm/s
- Stroke length: 4 mm (total distance traveled in one full cycle)
- Load: 25 N (this will not affect the distance calculation but is noted for context)
- Duration: 60 minutes = 3600 seconds

Step 1: Calculate the Number of Cycles

The stroke length is 4 mm, which means that in each forward and backward motion, the total distance traveled per cycle is:

Distance per cycle=4 mm(forward and backward movement)

The total distance traveled during the entire duration can be calculated using the sliding speed and the time:

Total distance traveled=Sliding speed×Time

Calculating the total distance traveled in 60 minutes:

Total distance traveled=2 mm/s×3600 s=7200 mm=7.2 meters

Step 2: Calculate the Number of Cycles

To find the total number of cycles, divide the total distance traveled by the distance per cycle:

Number of cycles=Total distance traveled/Distance per cycle=7200 mm/4 mm=1800 cycles

Step 3: Calculate Frequency

The frequency can be determined from the number of cycles over the duration in seconds:

Frequency=Number of cyclesTotal time in seconds=1800 cycles/3600 s=0.5 Hz

Summary:

- Total distance traveled: 7.2 meters
- Frequency: 0.5 Hz

**Li 2016 [37]**

Given

1. Contact Area:
   - Diameter of Cartilage Pin: 9 mm
   - Radius: r=9 mm2=4.5 mmr=29mm​=4.5mm (assumed to be stroke length as no other information was provided)

- Stroke Length LL: 4.5 mm
- Distance per cycle:
- Dcycle=2×L=2×4.5 mm=9 mm
- Calculating Frequencies for Each Velocity:
  - For 10 mm/s:
    f10=10 mm/s9 mm≈1.11 Hz
  - For 20 mm/s:
    f20=20 mm/s9 mm≈2.22 Hz
- Frequency at 10 mm/s: 1.11 Hz
- Frequency at 20 mm/s: 2.22 Hz

### Total Distance Traveled Calculation

1. For 10 mm/s:
   - Total time (2 hours = 7200 seconds):
2. Total Cycles=f10×Total Time=1.11 Hz×7200 s≈7992 cycles
   - Total distance traveled:
3. Dtotal,10=Dcycle×Total Cycles=9 mm×7992≈71928 mm≈71.93 meters
4. For 20 mm/s:
   - Total Cycles:
5. Total Cycles=f20×Total Time=2.22 Hz×7200 s≈15984 cycles

Total distance traveled:

Dtotal,20=Dcycle×Total Cycles=9 mm×15984≈143856 mm≈143.86 meters

### Summary of Distances Traveled

- Distance traveled at 10 mm/s: 71.93 meters
- Distance traveled at 20 mm/s: 143.86 meters

**Lizhang 2011 [38]**

1. Combination 1: Stroke Length = 4 mm, Sliding Velocity = 4 mm/s
2. Combination 2: Stroke Length = 8 mm, Sliding Velocity = 8 mm/s

Calculate these for durations of 1 hour (3600 seconds) and 24 hours (86,400 seconds).

1. Combination 1: Stroke Length = 4 mm, Sliding Velocity = 4 mm/s

For 1 Hour:

- Total Distance Traveled:

Total Distance=Sliding Speed×Time=4 mm/s×3600 s=14,400 mm=14.4 meters

Number of Cycles:

Number of Cycles=Total DistanceStroke Length=14,400 mm4 mm=3,600 cycles

Frequency:

Frequency=Number of CyclesTotal Time=3,600 cycles3600 s=1 HzF

For 24 Hours:

- Total Distance Traveled:

Total Distance=4 mm/s×86,400 s=345,600 mm=345.6 meters

Number of Cycles:

Number of Cycles=345,600 mm4 mm=86,400 cycles

Frequency:

Frequency=86,400 cycles86,400 s=1 Hz

2. Combination 2: Stroke Length = 8 mm, Sliding Velocity = 8 mm/s

For 1 Hour:

- Total Distance Traveled:

Total Distance=8 mm/s×3600 s=28,800 mm=28.8 metersTotal Distance=8mm/s×3600s=28,800mm=28.8meters

- Number of Cycles:

Number of Cycles=28,800 mm/8 mm=3,600 cycles

Frequency:

Frequency=3,600 cycles3600 s=1 Hz

For 24 Hours:

- Total Distance Traveled:

Total Distance=8 mm/s×86,400 s=691,200 mm=691.2 meters

Number of Cycles:

Number of Cycles=691,200 mm/8 mm=86,400 cycles

Frequency:

Frequency=86,400 cycles86,400 s=1 Hz

Summary of Results:

Combination 1: Stroke Length = 4 mm, Sliding Velocity = 4 mm/s

- For 1 Hour:
  - Total Distance Traveled: 14.4 meters
  - Frequency: 1 Hz
- For 24 Hours:
  - Total Distance Traveled: 345.6 meters
  - Frequency: 1 Hz

Combination 2: Stroke Length = 8 mm, Sliding Velocity = 8 mm/s

- For 1 Hour:
  - Total Distance Traveled: 28.8 meters
  - Frequency: 1 Hz
- For 24 Hours:
  - Total Distance Traveled: 691.2 meters
  - Frequency: 1 Hz

**Lizhang 2013 [52]**

Given Data:

- Flexion and Extension Angle: ±15° (30° total motion)
- Frequency: 1 Hz (1 cycle per second)
- Duration: 2 hours = 7200 seconds
- Diameters: 32 mm, 34 mm, 35 mm, 36 mm, and 37 mm

Step 1: Calculate Radius for Each Diameter

The radius rr is half the diameter:

- For 32 mm: r=322=16 mmr=232​=16mm
- For 34 mm: r=342=17 mmr=234​=17mm
- For 35 mm: r=352=17.5 mmr=235​=17.5mm
- For 36 mm: r=362=18 mmr=236​=18mm
- For 37 mm: r=372=18.5 mmr=237​=18.5mm

Step 2: Convert the Flexion and Extension Angle to Radians

Convert the angle from degrees to radians:

θ=15°×π180≈0.2618 radiansθ=15°×180π​≈0.2618radians

Step 3: Calculate the Arc Length per Cycle

The arc length LL for a given angle of flexion and extension can be calculated using the formula:

L=r×θL=r×θ

The total arc length for one complete cycle (forward and backward movement) is:

Arc Length per cycle=2×(r×θ)

Step 4: Calculate Total Distance Traveled, Number of Cycles, and Velocity for Each Diameter

1. Diameter = 32 mm

- Radius: 16 mm
- Arc Length per Cycle:L=2×(16 mm×0.2618)≈8.38 mm

Total Distance Traveled:

- Total Distance=8.38 mm×7200 cycles≈60,336 mm≈60.34 meters
- Number of Cycles:7200 cycles
- Velocity:Velocity=60,336 mm7200 s≈8.38 mm/s

2. Diameter = 34 mm

- Radius: 17 mm
- Arc Length per Cycle:L=2×(17 mm×0.2618)≈8.90 mm
- Total Distance Traveled:
  - Total Distance=8.90 mm×7200 cycles≈64,080 mm≈64.08 meters
- Number of Cycles:7200 cycles
- Velocity:
  - Velocity=64,080 mm7200 s≈8.90 mm/s

3. Diameter = 35 mm

- Radius: 17.5 mm
- Arc Length per Cycle:L=2×(17.5 mm×0.2618)≈9.15 mm
- Total Distance Traveled:Total Distance=9.15 mm×7200 cycles≈65,880 mm≈65.88 meters
- Number of Cycles:7200 cycles
- Velocity:Velocity=65,880 mm7200 s≈9.15 mm/s
- Diameter = 36 mm
- Radius: 18 mm18mm
- Arc Length per Cycle:L=2×(18 mm×0.2618)≈9.41 mmL=2×(18mm×0.2618)≈9.41mm
- Total Distance Traveled:
- Total Distance=9.41 mm×7200 cycles≈67,272 mm≈67.27 meters
- Number of Cycles:7200 cycles
- Velocity:Velocity=67,272 mm7200 s≈9.41 mm/s
- 5. Diameter = 37 mm
- Radius: 18.5 mm18.5mm
- Arc Length per Cycle:L=2×(18.5 mm×0.2618)≈9.67 mm
- Total Distance Traveled:Total Distance=9.67 mm×7200 cycles≈69,072 mm≈69.07 meters
- Number of Cycles:7200 cycles
- Velocity:Velocity=69,072 mm7200 s≈9.67 mm/sV
- Summary of Results for Each Diameter:

1. Diameter: 32 mm
   - Total Distance Traveled: 60.34 meters
   - Total Number of Cycles: 7200 cycles
   - Velocity: 8.38 mm/s
2. Diameter: 34 mm
   - Total Distance Traveled: 64.08 meters
   - Total Number of Cycles: 7200 cycles
   - Velocity: 8.90 mm/s
3. Diameter: 35 mm
   - Total Distance Traveled: 65.88 meters
   - Total Number of Cycles: 7200 cycles
   - Velocity: 9.15 mm/s
4. Diameter: 36 mm
   - Total Distance Traveled: 67.27 meters
   - Total Number of Cycles: 7200 cycles
   - Velocity: 9.41 mm/s
5. Diameter: 37 mm
   - Total Distance Traveled: 69.07 meters
   - Total Number of Cycles: 7200 cycles
   - Velocity: 9.67 mm/s

To calculate the average of 8.90 mm/s and 9.15 mm/s, you can use the formula for the average:

Average=Value 1+Value 2

Substituting in the values:

Average=8.90 mm/s+9.15 mm/s2=18.05 mm/s2=9.025 mm/s

Average Velocity: 9.03 mm/s (rounded to two decimal places) for medium group in the COF tabulations and calculations, L, XL were then used as 9.41, and 9.67 and small was 8.38

**LU 2023 [39]**

Given:

- Diameter: 6.36 mm
- Radius: r=6.362=3.18 mm=0.00318 m
- Sliding Velocity: 10 mm/s=0.01 m/s
- Stroke Length: 20 mm=0.02 m

1. Distance Traveled for 180 Minutes

Reciprocal Motion Distance

- Total Time:180 minutes=180×60=10,800 s
- Distance Traveled:
- Distancereciprocal=SlidingVelocity×Time=0.01 m/s×10,800 s=108 m

Cross-Shear Motion Distance

- Stroke Length: 0.02 m
- Time per Cycle:Time per Cycle=2×Stroke LengthSliding Velocity=2×0.02 m0.01 m/s=4 s
- Time per Cycle=Sliding
- Number of Cycles:Number of Cycles180=10,800 s4 s=2,700 cycles
- Distance per Cycle:
- Distancecycle=2πr=2π(0.00318)≈0.01996 m

Total Distance

- Distancecross-shear=Distancecycle×Number of Cycles180=0.01996 m×2,700≈53.91 m

Total Distance for 180 Minutes

Total Distance180=Distancereciprocal+Distancecross-shear=108 m+53.91 m≈161.91 m

2. Distance Traveled for 90 Minutes

Reciprocal Motion Distance

- Total Time:90 minutes=90×60=5,400 s
- Distance Traveled:Distancereciprocal=0.01 m/s×5,400 s=54 m

Cross-Shear Motion Distance

- Number of Cycles:Number of Cycles90=5,400 s4 s=1,350 cycles
- Total Distance:Distancecross-shear=Distancecycle×Number of Cycles90=0.01996 m×1,350≈26.96 m
- Total Distance for 90 Minutes

Total Distance90=Distancereciprocal+Distancecross-shear=54 m+26.96 m≈80.96 m

Frequency Calculations

1. For 180 Minutes

- Number of Cycles: 2,700 cycles
- Total Time: 180 minutes = 180×60=10,800 s

Frequency180=2,700 cycles10,800 s≈0.25 Hz

2. For 90 Minutes

- Number of Cycles: 1,350 cycles
- Total Time: 90 minutes = 90×60=5,400 s

Frequency90=1,350 cycles5,400 s≈0.25 Hz

**Luo 2010 [40]**

Given:

- Angular range: From -10° to 13.1° (extension-flexion)
  - Total angular range = 13.1∘−(−10∘)=23.1∘13.1∘−(−10∘)=23.1∘
- Number of cycles: 3600 cycles (at 1 Hz, meaning 1 cycle per second over 1 hour)
- Radius range: 38 mm to 42 mm
  - r1=38 mm=0.038 m
  - r2=42 mm=0.042 m

1. Angular Range:
   The total angle of motion is 23.1°.
2. Convert the angle to radians:
   θ=23.1∘×π180∘=0.403 radians
3. Calculate the arc length for one cycle:
   The distance traveled in one cycle is the arc length given by the formula:
   s=r×θs=r×θ
   - For the smaller radius (38 mm = 0.038 m):
     s1=0.038 m×0.403 rad=0.015314 m
   - For the larger radius (42 mm = 0.042 m):
     s2=0.042 m×0.403 rad=0.016926 m

Distance for One Full Cycle (Reciprocating Motion):
In reciprocating motion, one full cycle includes both a forward and backward stroke. So, the total distance for one cycle is twice the arc length:

- For the smaller radius:
  Distance per cycle1=2×0.015314 m=0.030628 m
- For the larger radius:
  Distance per cycle2=2×0.016926 m=0.033852 m

Total Distance for 3600 Cycles: Now, multiply the distance per cycle by the number of cycles (3600):

- For the smaller radius:
  Total Distance1=0.030628 m×3600=110.26 m
- For the larger radius:
  Total Distance2=0.033852 m×3600=121.87 m

Velocity Calculation: Since each cycle takes 1 second (due to the frequency being 1 Hz), the velocity is the total distance traveled per cycle divided by the time per cycle (which is 1 second):

Velocity1=Distance per cycle1/1 second=0.030628 m/s

Velocity2=Distance per cycle2/1 second=0.033852 m/s

Velocity in mm/s:

- For the smaller radius (38 mm):
  Velocity1=0.030628 m/s×1000=30.63 mm/sVelocity1​=0.030628m/s×1000=30.63mm/s
- For the larger radius (42 mm):
  Velocity2=0.033852 m/s×1000=33.85 mm/sVelocity2​=0.033852m/s×1000=33.85mm/s

The velocity for the reciprocating motion ranges from 30.63 mm/s to 33.85 mm/s, depending on the radius.

The total distance traveled over 3600 cycles with reciprocating motion (forward and backward) for an angular range of -10° to 13.1° and radii ranging from 38 mm to 42 mm is between 110.26 meters and 121.87 meters.

**McCann 2008 [53]**

Given:

- Flexion-extension range: From -10.1° to 13.11°
  - Total angular range = 13.11∘−(−10.1∘)=23.21∘13.11∘−(−10.1∘)=23.21∘
- Radius (approximate width of the surface): 22 mm = 0.022 m
- Cycles:
  - 3600 cycles at 1 Hz (1 hour)
  - 300 cycles at 1 Hz (high loads)
- Frequency: 1 Hz (1 cycle per second)

Steps:

1. Convert the angular range to radians:

θ=23.21∘×π180∘=0.405 radians

2. Calculate the arc length (distance per stroke):

For each cycle, the flexion-extension motion forms an arc. The arc length ss is given by:

s=r×θs=r×θ

where rr is the radius.

Substitute r=0.022 mr=0.022m and θ=0.405 radians

s=0.022 m×0.405 rad=0.00891 m=8.91 mms

This is the arc length for one stroke.

3. Distance per full reciprocating cycle:

In a reciprocating motion, a full cycle includes a forward and a backward stroke. So, the total distance per cycle is:

Distance per cycle=2×8.91 mm=17.82 mm

4. Calculate the total distance for both scenarios:

- For 3600 cycles:
  Total distance=3600×17.82 mm=64,152 mm=64.152 meters
- For 300 cycles (high loads):
  Total distance=300×17.82 mm=5,346 mm=5.346 meters

5. Calculate the velocity:

Velocity is the total distance traveled per unit time. Since we are given a frequency of 1 Hz, each cycle takes 1 second. Thus, the velocity is the distance per cycle.

Velocity=Distance per cycleTime per cycle=17.82 mm1 s=17.82 mm/s

Final Answers:

- The total distance traveled for 3600 cycles is 64.152 meters.
- The total distance traveled for 300 cycles (high loads) is 5.346 meters.
- The velocity is 17.82 mm/s.

**McCann 2009 [54]**

The friction simulator methodology has been described in previous study (McCann et al., 2008).

**Morimoto 2014 [41]**

Unidirectional - no frequency or number of cycles

Given:

Total distance: 144 m

Velocity: 20mm/s

**NORTHWOOD 2007 [25]**

Given:

- Linear (reciprocating) motion:
  - Stroke length = 10 mm (forward and backward per cycle)
  - Sliding speed = 4 mm/s
  - Duration = 8 hours = 28,800 cycles
- Circular motion (multi-directional):
  - Rotation angle = ±10° = 20° (total arc angle)
  - Pin diameter = 8 mm
  - Number of cycles = 28,800

1. Linear Reciprocating Motion Distance

- Each cycle involves a forward and backward motion, meaning the stroke length needs to be doubled per cycle.
  Distance per cycle=2×10mm=20mm
- Total distance for 28,800 cycles:
  Total linear distance=20mm×28,800cycles=576,000mm=576meters

2. Circular Motion (Rotational) Distance

- First, calculate the radius from the pin diameter:
  Radius=8 mm2=4 mmRadius=28mm​=4mm
- Convert the angle from degrees to radians (since arc length is calculated using radians):
  Angle in radians=180∘20∘×π​=0.3491radians
- The distance traveled per cycle due to rotational motion (arc length):
  Arc length per cycle=Radius×Angle=4 mm×0.3491=1.3964 mm
- Total distance for 28,800 cycles:
  Total circular distance=1.3964 mm×28,800 cycles=40,186 mm=40.186 meters

3. Total Distance Traveled

- Now, sum the distances from both the linear and circular motions:
  Total distance=576 m+40.186 m=616.186 meters

4. Frequency

- The frequency is the number of cycles per second. Given that the test was run for 8 hours (28,800 seconds) with 28,800 cycles, the frequency is:
- Frequency=Total Time (s)/Number of Cycles​
- Frequency=28,80028,800=1 Hz

Summary:

- Total distance traveled: 616.186 meters over 8 hours, accounting for both reciprocating linear and multi-directional circular motion.
- Number of cycles: 28,800 cycles.
- Frequency: 1 Hz

**Northwood 2007 [42]**

### Given Data:

- Stroke Length: 10 mm = 0.01 m (using stroke length from Northwood 2007 above as this article did not provide but as they have the same set up this was assumed)
- Frequency (f): 0.2 Hz
- Cycles in 8 hours: 28,800 cycles

### 8 Hours:

1. Time in seconds:8 hours=28800 s
2. Total Cycles:Total cycles=28800 cycles
3. Total Distance Traveled:
   1. Distance=Total cycles×Stroke Length×2=28800×0.01×2=576 m

### 4 Hours:

1. Time in seconds:4 hours=14400 s
2. Total Cycles:Total cycles=0.2 Hz×14400 s=2880 cycles
3. Total Distance Traveled:Distance=Total cycles×Stroke Length×2=2880×0.01×2=57.6 m

### Summary of Distances Traveled:

- 4 hours: 57.6 m (2880 cycles)
- 8 hours: 576 m (28,800 cycles)

Note 0.5 Mpa was used from Northwood 2007 Covidence 171 as this was not explicitly stated but same testing set up as the prior entry

Oungoulian 2015

Given:

- Stroke length: ±5.0 mm (the sliding motion moves 5 mm in one direction and then 5 mm back, making a total distance of 10 mm per cycle)
- Velocity: 1 mm/s
- Test duration: 4 hours = 4 × 60 × 60 = 14,400 seconds

Steps:

1. Distance per cycle:

Since the motion is reciprocating (forward and backward):

Distance per cycle=2×5.0 mm=10.0 mm

2. Time per cycle:

To find the time for each full cycle (forward and backward), use the velocity:

Time per cycle=Distance per cycleVelocity=10.0 mm1.0 mm/s=10.0 seconds

3. Total number of cycles:

For a test lasting 14,400 seconds, the number of cycles is:

Number of cycles=14,400 seconds/10.0 seconds per cycle=1,440 cycles

4. Total distance traveled:

To find the total distance, multiply the distance per cycle by the number of cycles:

Total distance=1,440×10.0mm=14,400mm=14.4meters

5. Time per cycle: 10 seconds (as calculated earlier, since it takes 10 seconds to complete one full cycle, which includes forward and backward movement)

Calculate frequency:

Frequency=110 seconds per cycle=0.1 Hz

Final Answer:

- The total number of cycles during the 4-hour test is 1,440 cycles.
- The total distance traveled is 14.4 meters.
- The velocity is 1 mm/s as given.

**Patel 1997 [44]**

Given:

- Radius of disc: 7 mm (or 0.007 m)
- Revolutions per km: 22,736 rev/km
- Total distance: 9 km
- Speed: 0.1 m/s (or 0.0001 km/s)

Step 1: Calculate the Circumference of the Disc

The circumference CC of the disc is given by:

C=2πrC=2πr

Substituting r=0.007 mr=0.007m:

C=2π×0.007≈0.04396 m

Step 2: Calculate the Total Distance Traveled by the Disc

First, find the total number of revolutions for the entire 9 km:

Total revolutions=22,736 rev/km×9 km=204,624 revolutions

Then, calculate the total distance traveled by the disc:

Total distance traveled by disc=Total revolutions×C

Total distance traveled by disc=204,624 revolutions×0.04396 m/rev≈9,000 m

Final Answer:

The disc traveled approximately 9,000 meters (or 9 km), confirming that the distance matches the total slide distance.

Step 3: Calculate the Total Duration of the Study

The time tt to cover a distance is given by:

t=DistanceSpeed=9 km0.0001 km/s=90,000 seconds

Step 4: Calculate Frequency in Revolutions per Second

To find the frequency ff:

f=Revolutions per kmTime per km (s)f=Time per km (s)Revolutions per km​

First, calculate the time to cover 1 km at the given speed:

Time to cover 1 km=1 km0.0001 km/s=10,000 s/kmTime to cover 1 km=0.0001km/s1km​=10,000s/km

Then, substitute into the frequency equation:

The frequency is approximately 2.2736 Hz (revolutions per second).

Step 5: Calculate Total Number of Cycles (Revolutions) for the Pin

The total number of cycles (revolutions) is:

Total cycles=Revolutions per km×Total distance (km)

Total cycles=22,736 rev/km×9 km=204,624 revolutions

The total number of cycles is 204,624 revolutions.

Summary

The calculations indicate that the disc traveled 9,000 meters (or 9 km). The frequency of motion is approximately 2.2736 Hz, and the total number of revolutions is 204,624 revolutions. The analysis remains consistent across continuous rotational sliding scenarios.

**Qian 2019 [45]**

Given:

- Sliding velocity: 2 mm/s
- Sliding length (one way): 10 mm
- Total sliding time: 3600 seconds

Steps:

1. Total distance traveled:

For one complete cycle (forward and backward), the total sliding length is actually 20 mm:

Total sliding length per cycle=2×10 mm=20 mmTotal sliding length per cycle=2×10mm=20mm

To find the total distance traveled during the entire sliding time, we can use the formula:

Distance=Velocity×TimeDistance=Velocity×Time

However, since the sliding length for each complete cycle is 20 mm, we will calculate the number of cycles first.

2. Number of cycles:

First, calculate the number of cycles in 3600 seconds:

Time per cycle=Sliding length (two-way)Velocity=20 mm2 mm/s=10 seconds

Now, calculate the total number of cycles during the 3600 seconds:

Number of cycles=3600 s10 s/cycle=360 cycles

3. Total distance traveled:

Now, calculate the total distance using the distance per complete cycle:

Total distance=Number of cycles×Sliding length (two-way)=360×20 mm=7200 mm=7.2 meters

4. Frequency:

Frequency=1/Time per cycle=1/10 s=0.1 Hz

Final Answers:

- Total distance traveled: 7.2 meters
- Frequency: 0.1 Hz

**Sardinha 2013 [46]**

Note that within the text they say that they traveled 500 cycles but this does not reflect based on their frequency provided and duration of the test, for consistency across our calculations we focused on calculating the number of cycles based on the following parameters

Given:

- Frequency: 1 Hz (1 cycle per second)
- Stroke length: 8 mm (each direction in the reciprocating motion)
- Total time: 2 hours = 120 minutes = 7,200 seconds

1. Velocity:

- Since the stroke length is 8 mm, each full cycle (back and forth) is:Full cycle distance=2×8 mm=16 mm
- At 1 Hz, there is 1 full cycle per second.
- Therefore, the velocity is:Velocity=Distance per cycle/Time per cycle=16 mm/1 second=16 mm/s

2. Number of cycles:

- Since the frequency is 1 cycle per second, the number of cycles in 7,200 seconds is:Number of cycles=1 Hz×7,200 seconds=7,200 cyclesNumber of cycles=1Hz×7,200seconds=7,200cycles

3. Total distance traveled:

- Each full cycle covers 16 mm, so the total distance traveled is:Total distance=7,200cycles×16mm=115,200mm=115.2meters

Summary:

- Velocity: 16 mm/s
- Number of cycles: 7,200 cycles
- Total distance traveled: 115.2 meters

**Wan 2020 [47]**

Given:

- Sliding speed: 4 mm/s
- Stroke length: 10 mm (one-way distance)
- Total time: 60 minutes = 3,600 seconds

1. Time per cycle:

- The sliding distance for a full cycle (back and forth) is 20 mm (since each stroke is 10 mm).
- The velocity is 4 mm/s, so the time taken for each stroke (one-way) is:Time per stroke=10 mm4 mm/s=2.5 seconds
- Therefore, the time for a full cycle (back and forth) is:Time per cycle=2×2.5 seconds=5 seconds

2. Number of cycles:

- The number of cycles in 60 minutes is:
- Number of cycles=3,600 seconds/5 seconds per cycle=720 cycles

3. Total distance traveled:

- Each full cycle covers 20 mm, so the total distance traveled is:Total distance=720 cycles×20 mm=14,400 mm=14.4 meters

4. Frequency

- Frequency is the reciprocal of the time per cycle:
- Frequency=Time per cycle=1/5 seconds=0.2 Hz

Summary:

- Total distance traveled: 14.4 meters in 60 minutes

This end total distance travels is not what the author states “a sliding speed of 4 mm/s. The sliding distance used was 10 mm per cycle, which gave a total distance of 1.44 m in 60 min of sliding.” I believe that this is a type based on the above calculations. Also the next study Wan 2021 with the same inputs they report the same outputs that match these calculations.

**Wan 2021 [48]**

Given:

- Sliding speed: 4 mm/s
- Sliding distance per stroke: 10 mm (one-way distance)
- Total time: 1 hour = 3,600 seconds
- Number of cycles: 720 cycles

1. Total distance traveled:

Since each full cycle consists of moving 10 mm in one direction and 10 mm in the opposite direction (totaling 20 mm per cycle), calculate the total distance traveled:

Total distance=Number of cycles×Distance per cycle

Distance per cycle=10 mm (forward)+10 mm (backward)=20 mm

Total distance=720 cycles×20 mm/cycle=14,400 mm=14.4 meters

2. Frequency:

The frequency can be calculated as follows:

Frequency=Number of cycles/Total time in seconds=720 cycles/3600 seconds=0.2 Hz

Summary:

- Total distance traveled: 14.4 meters
- Frequency: 0.2 Hz

**Zhang 2021 [49]**

Given:

- Speed: 2 mm/s
- Time: 0.5 hours

Detailed Calculation:

1. Convert Time from Hours to Seconds:
   Time (seconds)=Time (hours)×3600Time
2. Time (seconds)=0.5hours×3600seconds/hour=1800seconds
3. Calculate Distance Traveled:
   Distance (mm)=Speed (mm/s)×Time (seconds)
4. Distance (mm)=2 mm/s×1800 seconds=3600 mmDistance (mm)=2mm/s×1800seconds=3600mm
5. Convert Distance to Meters:
   Distance (meters)=Distance (mm)/1000D
6. sDistance (meters)=3600mm/1000​=3.6meters

Summary:

- Distance Traveled:
  - In millimeters: 3600 mm
  - In meters: 3.6 m

Given:

- Speed: 2 mm/s
- Time: 12 hours

Detailed Calculation:

1. Convert Time from Hours to Seconds:
   Time (seconds)=Time (hours)×3600Time (seconds)
2. Time (seconds)=12hours×3600seconds/hour=43200seconds
3. Calculate Distance Traveled:
   Distance (mm)=Speed (mm/s)×Time (seconds)
4. Distance (mm)=2 mm/s×43200 seconds=86400 mm
5. Convert Distance to Meters:
   Distance (meters)=Distance (mm)/1000
6. Distance (meters)=86400mm/1000​=86.4meters

Summary:

- Distance Traveled:
  - In millimeters: 86400 mm
  - In meters: 86.4 m
