# Supplementary material for: Friction and Cartilage Wear in Hemiarthroplasty: A Systematic Review of Key Influencing Factors
Source: Lubricants. Author manuscript; Available in PMC 2026 Feb 11. (PMC12889880; doi:10.3390/lubricants14010018)
Supplement: Suppl Material 4 [file NIHMS2141622-supplement-Suppl_Material_4.docx]

**Supplementary Material 4. COF Predictive Modelling**

This supplementary material provides the results of coefficient of friction (COF) predictive modeling across all available hemiarthroplasty bearing material (HBM)–lubricant combinations. Linear regression and stepwise regression models were developed wherever possible, and summary tables of model coefficients, performance metrics, and statistical tests are presented for each dataset. Our goal was to maximize the inclusion of models to capture potential trends in COF as a function of time, contact stress, and sliding velocity. However, in certain experimental subsets, predictor variables lacked variability (for example, when velocity was held constant across all replicates), which limited the ability to build robust predictive models. In such cases, models are either omitted as the absence of variation restricts their predictive utility. The following tables are organized by HBM and then by lubricant for clarity.

| Model |
| --- |
| COF ~ 1 + AvgTime_min + Contactstress + Velocity |

**Supplementary Table 18. Hydrogel-BCS Full Model**

| Coefficient | Estimate | SE | tStat | pValue |
| --- | --- | --- | --- | --- |
| (Intercept) | 2.606452145 | 12.20021339 | 0.213639882 | 0.834142051 |
| AvgTime_min | -0.00543939 | 0.030245195 | -0.179843101 | 0.860048766 |
| Contactstress | -2.684113732 | 13.9895301 | -0.191865896 | 0.850811738 |
| Velocity | 0.017745604 | 0.139878218 | 0.126864668 | 0.900988773 |

| Observations | R_squared | Adj_R_squared | RMSE | F_stat | pValue |
| --- | --- | --- | --- | --- | --- |
| 17 | 0.7748011 | 0.72283209 | 0.05 | 14.91 | 0.0002 |

**Supplementary Table 19. Hydrogel-BCS Backward Elimination Model**

| Coefficient | Estimate | SE | tStat | pValue |
| --- | --- | --- | --- | --- |
| (Intercept) | 1.058805065 | 0.151844157 | 6.97297205 | 6.52132E-06 |
| AvgTime_min | -0.001602664 | 0.000377146 | -4.2494493 | 0.000809008 |
| Contactstress | -0.909438897 | 0.144801301 | -6.28059895 | 2.02009E-05 |

| Observations | R_squared | Adj_R_squared | RMSE | F_stat | pValue |
| --- | --- | --- | --- | --- | --- |
| 17 | 0.7745223 | 0.74231116 | 0.05 | 24.05 | 3E-05 |

**Supplementary Table 20. HXLPE-BCS Full Model**

| Coefficient | Estimate | SE | tStat | pValue |
| --- | --- | --- | --- | --- |
| (Intercept) | 0 | 0 |  |  |
| AvgTime_min | -0.028702412 | 0.010094186 | -2.843459705 | 0.215288559 |
| Contactstress | -0.035284717 | 0.004600013 | -7.670569822 | 0.082529653 |
| Velocity | 0.123446097 | 0.033710887 | 3.661905908 | 0.169711397 |

| Observations | R_squared | Adj_R_squared | RMSE | F_stat | pValue |
| --- | --- | --- | --- | --- | --- |
| 5 | 0.9724057 | 0.944811449 | 0.005 | 11.75 | 0.2105 |

**Supplementary Table 21. HXLPE-BCS Backward Elimination Model**

| Coefficient | Estimate | SE | tStat | pValue |
| --- | --- | --- | --- | --- |
| (Intercept) | 0.2764738 | 0.021280123 | 12.992113 | 0.000984568 |
| Contactstress | -0.0362386 | 0.008411707 | -4.3081103 | 0.023024453 |

| Observations | R_squared | Adj_R_squared | RMSE | F_stat | pValue |
| --- | --- | --- | --- | --- | --- |
| 5 | 0.8608522 | 0.814469649 | 0.012 | 18.56 | 0.023 |

**Supplementary Table 22. PCU-BCS Full Model**

| Coefficient | Estimate | SE | tStat | pValue |
| --- | --- | --- | --- | --- |
| (Intercept) | -0.771120657 | 4.852167981 | -0.15892291 | 0.899665616 |
| AvgTime_min | -0.118212801 | 0.605757114 | -0.195148845 | 0.877306394 |
| Contactstress | 0 | 0 |  |  |
| Velocity | 1.233039021 | 6.275486377 | 0.196485013 | 0.876487175 |

| Observations | R_squared | Adj_R_squared | RMSE | F_stat | pValue |
| --- | --- | --- | --- | --- | --- |
| 5 | 0.8160425 | 0.632085002 | 0.041 | 1.479 | 0.5289 |

**Supplementary Table 23. PCU-BCS Backward Elimination Model**

| Coefficient | Estimate | SE | tStat | pValue |
| --- | --- | --- | --- | --- |
| (Intercept) | 0.175732767 | 0.037466144 | 4.69044173 | 0.018321687 |
| Velocity | 0.008385228 | 0.002325341 | 3.60602076 | 0.036606253 |
| Contactstress | -0.909438897 | 0.144801301 | -6.28059895 | 2.02009E-05 |

| Observations | R_squared | Adj_R_squared | RMSE | F_stat | pValue |
| --- | --- | --- | --- | --- | --- |
| 5 | 0.8125397 | 0.75005289 | 0.041 | 13 | 0.0366 |

**Supplementary Table 24. PCU-PBS Full Model**

| Coefficient | Estimate | SE | tStat | pValue |
| --- | --- | --- | --- | --- |
| (Intercept) | 0 | 0 |  |  |
| AvgTime_min | -0.299884756 | 0.712813182 | -0.420705962 | 0.714865282 |
| Contactstress | -0.03133908 | 0.036719866 | -0.85346391 | 0.483308771 |
| Velocity | 2.571795166 | 6.001145017 | 0.428550745 | 0.709991873 |

| Observations | R_squared | Adj_R_squared | RMSE | F_stat | pValue |
| --- | --- | --- | --- | --- | --- |
| 6 | 0.2029453 | -0.328424436 | 0.086 | 0.17 | 0.9086 |

**Supplementary Table 25. PCU-PBS Backward Elimination Model**

| Coefficient | Estimate | SE | tStat | pValue |
| --- | --- | --- | --- | --- |
| (Intercept) | 0.141812 | 0 | 3.29 | 0.0218 |
| AvgTime_min | 0.000165 | 0 | 3.82 | 0.0012 |
| Velocity | -0.02612 | 0 | -4.42 | 0.0003 |

| Observations | R_squared | Adj_R_squared | RMSE | F_stat | pValue |
| --- | --- | --- | --- | --- | --- |
| 6 | 0 | 0 | 0.096 |  |  |

**Supplementary Table 26. PEEK-BCS Full Model**

| Coefficient | Estimate | SE | tStat | pValue |
| --- | --- | --- | --- | --- |
| (Intercept) | 0.020877972 | 0.017758497 | 1.175660978 | 0.2669615 |
| AvgTime_min | 6.20874E-06 | 0.000124125 | 0.050020171 | 0.961091311 |
| Contactstress | -0.009089886 | 0.007982116 | -1.138781427 | 0.281339681 |
| Velocity | 0.013133678 | 0.001680906 | 7.813452158 | 1.44694E-05 |

| Observations | R_squared | Adj_R_squared | RMSE | F_stat | pValue |
| --- | --- | --- | --- | --- | --- |
| 14 | 0.8737667 | 0.835896655 | 0.018 | 23.07 | 8E-05 |

**Supplementary Table 27. PEEK-BCS Backward Elimination Model**

| Coefficient | Estimate | SE | tStat | pValue |
| --- | --- | --- | --- | --- |
| (Intercept) | 0.00904749 | 0.008948497 | 1.0110625 | 0.331939366 |
| Velocity | 0.012154562 | 0.001446211 | 8.40441713 | 2.26137E-06 |
| Contactstress | -0.909438897 | 0.144801301 | -6.28059895 | 2.02009E-05 |

| Observations | R_squared | Adj_R_squared | RMSE | F_stat | pValue |
| --- | --- | --- | --- | --- | --- |
| 14 | 0.8547817 | 0.842680201 | 0.019 | 70.63 | 2E-06 |

**Supplementary Table 28. PEEK-PBS Full Model**

| Coefficient | Estimate | SE | tStat | pValue |
| --- | --- | --- | --- | --- |
| (Intercept) | 0 | 0 |  |  |
| AvgTime_min | -0.299884756 | 0.712813182 | -0.420705962 | 0.714865282 |
| Contactstress | -0.03133908 | 0.036719866 | -0.85346391 | 0.483308771 |
| Velocity | 2.571795166 | 6.001145017 | 0.428550745 | 0.709991873 |

| Observations | R_squared | Adj_R_squared | RMSE | F_stat | pValue |
| --- | --- | --- | --- | --- | --- |
| 6 | 0.2029453 | -0.328424436 | 0.086 | 0.17 | 0.9086 |

**Supplementary Table 29. PEEK-PBS Backward Elimination Model**

| Coefficient | Estimate | SE | tStat | pValue |
| --- | --- | --- | --- | --- |
| (Intercept) | 0.141812 | 0 | 3.29 | 0.0218 |
| AvgTime_min | 0.000165 | 0 | 3.82 | 0.0012 |
| Velocity | -0.02612 | 0 | -4.42 | 0.0003 |

| Observations | R_squared | Adj_R_squared | RMSE | F_stat | pValue |
| --- | --- | --- | --- | --- | --- |
| 6 | 0 | 0 | 0.096 |  |  |
